# Suboptimal resource allocation in changing environments constrains response and growth in bacteria

**DOI:** 10.15252/msb.202110597

**Published:** 2021-12-20

**Authors:** Rohan Balakrishnan, Roshali T de Silva, Terence Hwa, Jonas Cremer

**Affiliations:** ^1^ Department of Physics University of California at San Diego La Jolla CA USA; ^2^ Department of Biology Stanford University Stanford CA USA; ^3^ Division of Biological Sciences University of California at San Diego La Jolla CA USA

**Keywords:** cellular response, diauxie, environmental changes, growth optimality, resource allocation, Metabolism, Microbiology, Virology & Host Pathogen Interaction

## Abstract

To respond to fluctuating conditions, microbes typically need to synthesize novel proteins. As this synthesis relies on sufficient biosynthetic precursors, microbes must devise effective response strategies to manage depleting precursors. To better understand these strategies, we investigate the active response of *Escherichia coli* to changes in nutrient conditions, connecting transient gene expression to growth phenotypes. By synthetically modifying gene expression during changing conditions, we show how the competition by genes for the limited protein synthesis capacity constrains cellular response. Despite this constraint cells substantially express genes that are not required, trapping them in states where precursor levels are low and the genes needed to replenish the precursors are outcompeted. Contrary to common modeling assumptions, our findings highlight that cells do not optimize growth under changing environments but rather exhibit hardwired response strategies that may have evolved to promote fitness in their native environment. The constraint and the suboptimality of the cellular response uncovered provide a conceptual framework relevant for many research applications, from the prediction of evolution to the improvement of gene circuits in biotechnology.

## Graphical abstract

Analyses of how allocation of cellular resources to different genes shapes *Escherichia coli*'s response to changing nutrient conditions show that growth transitions are determined by the competition between genes that are directly required in the encountered conditions and those that are not.

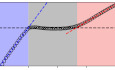

## Introduction

Changing environmental conditions are a hallmark of microbial habitats, and microbes have to respond appropriately to thrive (Stanier, 1951; Roszak & Colwell, 1987; Siegal, 2015; Bertrand, 2019; Erez *et al*, 2020; Moreno‐Gámez *et al*, 2020). For instance, the depletion of a preferred carbon source requires the efficient transitioning to the consumption of another carbon source (Monod, 1949, 1966). Several studies have characterized the response to such diauxic shifts by identifying the up‐ and downregulation of hundreds of genes (Chang *et al*, 2002; Kao *et al*, 2005; Mostovenko *et al*, 2011), and implicating major regulators such as cAMP (Loomis & Magasanik, 1967; Ullmann & Monod, 1968; Inada *et al*, 1996; Kimata *et al*, 1997) and ppGpp (Traxler *et al*, 2006; Fernández‐Coll & Cashel, 2018). Executing these different processes is a major challenge for the cell, especially when biosynthetic precursor levels drop during shifts, yet how cells navigate these challenges and strategize an optimal response remains poorly understood. To decipher the fundamental principles shaping the cellular response and growth kinetics, we here present a quantitative study on cell physiology connecting gene expression to growth phenotypes.

## Results

**Figure 1:**
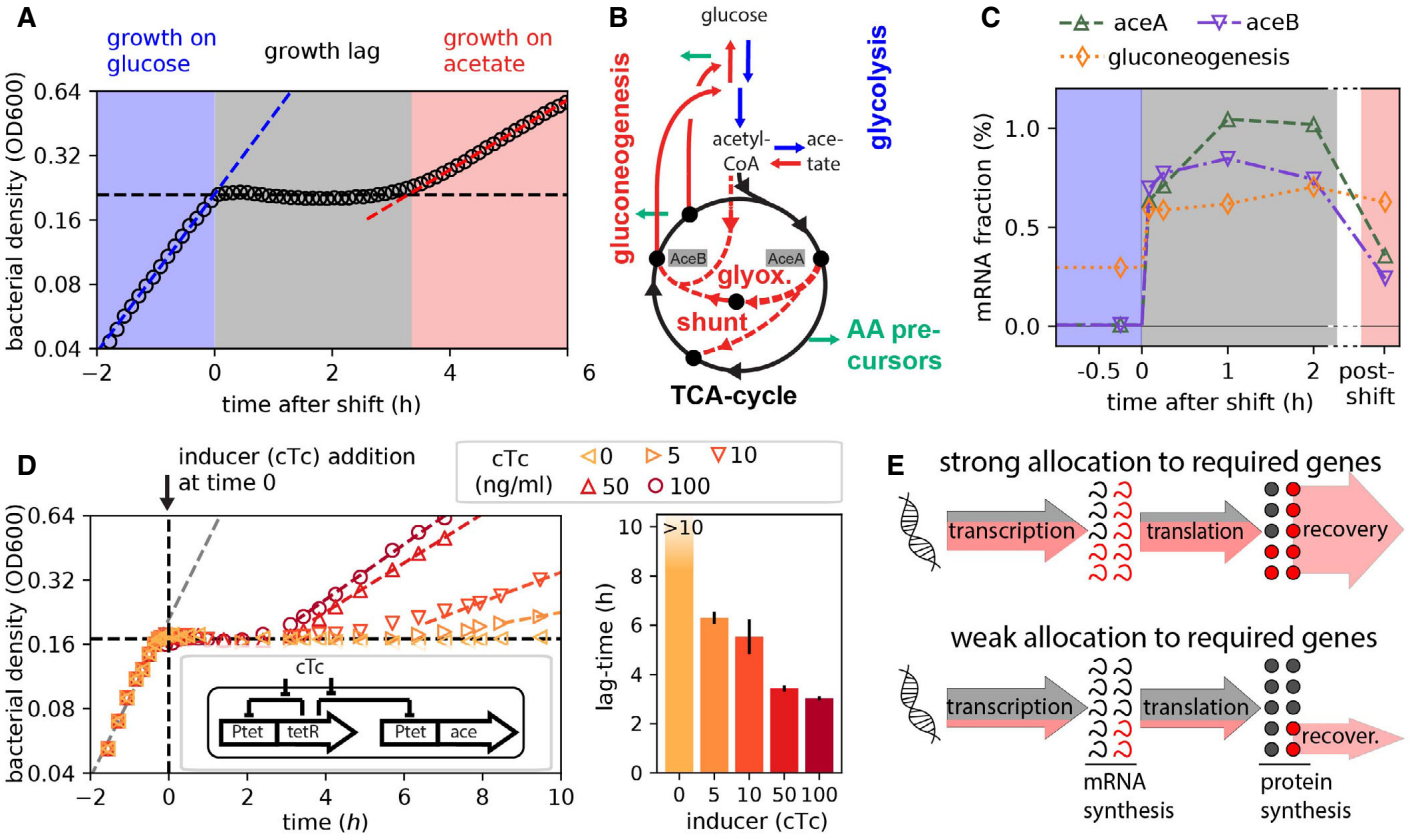
Diauxic shift from glucose to acetate Diauxic growth of WT *Escherichia coli* (NCM3722) in minimal media containing glucose and acetate, bacterial density measured as optical density (OD600). Growth on glucose is captured by the exponential fit (blue dashed line) and proceeds until glucose runs out (black dashed line) at time = 0 h. This is followed by a period of growth lag lasting ~3.5 h before exponential growth resumes on acetate (red dashed line).The central carbon metabolism pathways are illustrated along with the nodes branching out into amino acid precursor synthesis (green arrows). Glucose to acetate diauxie requires switching from the pathways facilitating glucose consumption (represented in blue) to those responsible for acetate consumption (represented in red). More details in Fig EV1B.The mRNA fractional abundances for aceB, aceA, and the gluconeogenesis genes (summed abundance of maeA, maeB, pck, and ppsA genes) were estimated by RNA sequencing performed at various time points during the diauxic transition as indicated on the *x*‐axis. The *x*‐axis is truncated 2.3 h into the shift, and the “post‐shift” (pink) regime represents transcript levels when growth fully resumes on acetate, measured under steady‐state growth on acetate minimal medium. The series of RNA‐Seq through the growth transition was performed once.Lag‐times for controlled titration of aceBA expression using an inducer construct in strain NQ1350 (inset). Addition of chlortetracycline (cTc) removes the tetR repression and induces aceBA expression. As the expression of aceB/aceA during the response (inducer added at time = 0 h) is increased, lag‐times decrease. Bar plot shows mean lag‐times (*N* = 3 biological repeats) for different inducer concentration with error bars denoting the standard deviations (SD).Of the overall transcription and translation fluxes (arrows), a strong allocation toward the expression of shunt and gluconeogenesis genes (red) increases the novel synthesis of required enzymes and should thus lead to faster growth recovery. Source data are available online for this figure.

**Figure EV1:**
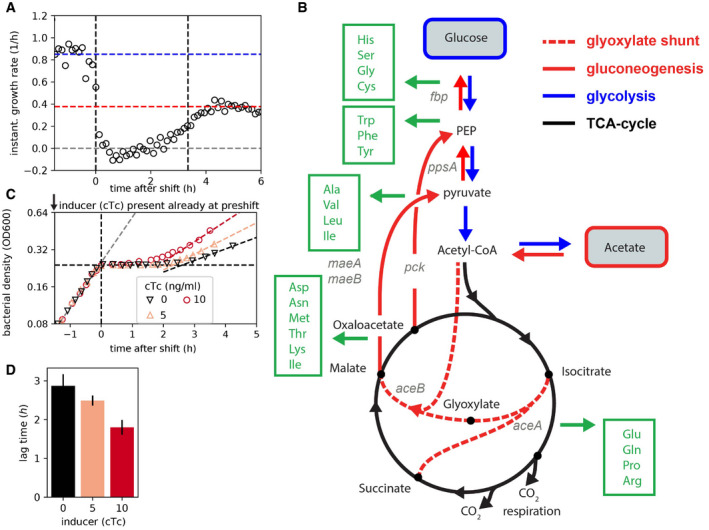
Diauxic growth on glucose and acetate: transition kinetics and metabolic requirements AInstantaneous growth rate of the WT (NCM3722) is derived by calculating the derivative of the growth rate divided by the optical density (growth curve shown in Fig 1A). Cells first consume glucose and grow exponentially at a rate of ~0.9/h (blue horizontal line). Following glucose depletion (defined as time 0; left vertical dashed line), the instantaneous growth rate immediately falls and growth stops. After a phase of no growth, growth gradually begins to approach a rate of 0.4/h, the steady‐state growth rate for growth on acetate (red horizontal line). Right vertical dashed line indicates the time of growth recovery as determined by the fitting of an exponential curve (see Lag‐time quantification in Materials and Methods).BTo resume growth on acetate following glucose depletion, the supply of amino acids, the major precursors required for biomass synthesis, must be re‐established. The various nodes along the central carbon metabolism that branch into the synthesis of different amino acids are indicated in green. For the successful recycling of the 2 carbon molecules per acetate into amino acid precursors, the most essential step is the activation of the glyoxylate shunt (Wolfe, 2005): To prevent the loss of the 2 CO_2_ molecules occurring along the TCA cycle, the carbon flux has to be bypassed. Instead of being converted to alpha‐KG, isocitrate is split into succinate and glyoxylate by the enzyme isocitrate lyase (*AceA*). Glyoxylate is then converted to malate by the malate synthase (*AceB*). Succinate and malate generated as a result of the shunt subsequently fuel gluconeogenesis (*MaeA*, *MaeB*, *Pck*, *PpsA*), making available the carbon precursors required for the synthesis of many amino acids (green arrows). Besides amino acids as precursors, protein synthesis also requires substantial amounts of energy, primarily to charge tRNA and drive translation. However, energy supply is unlikely to be the major bottleneck during this shift since cells already express and utilize TCA enzymes during the pre‐shift growth, which can thus ensure a continuous production of ATP over the course of the transition.C, DEffect of maintaining pre‐expressed aceBA reserves on growth transitions. Diauxic transitions of NQ1350 in which the native aceBA promoter is replaced by the inducible P*_tet_* promoter are shown when 0, 5, or 10 ng/ml inducer cTc is added to the medium already at the pre‐culture stage, well before glucose depletion. Bar plot (D) shows the mean lag‐times of *N* = 2 biological replicates. Error bars indicate SD. Source data are available online for this figure.

We first studied the shift from growth in glucose to growth in acetate, a shift previously used to study growth transitions (Kao *et al*, 2005; Kotte *et al*, 2014; Enjalbert *et al*, 2015; Basan *et al*, 2020). We grew *Escherichia coli*–K‐12 cells in batch cultures and tracked growth by measuring optical density (Fig 1A). The shift from growth on glucose (blue zone) to acetate (red zone) is accompanied by a period of growth arrest (lag‐time *τ_lag_*) lasting ~3.5 h (gray zone). The lag is also illustrated by the drop of the instantaneous growth rate during the shift (Fig EV1A). From the metabolic perspective, this transition requires a switch from glycolytic pathways to the activation of the glyoxylate shunt and gluconeogenesis pathways(Oh *et al*, 2002; Kao *et al*, 2005; Wolfe, 2005; Enjalbert *et al*, 2015) so that the synthesis of amino acids and other growth precursors (green arrows) can continue (Figs 1B and EV1B). Hence, following glucose depletion, the synthesis of the glyoxylate shunt enzymes (AceB, AceA) and gluconeogenesis enzymes (MaeA, MaeB, Pck, PpsA) is required before growth can resume on acetate (Fig EV1B). Indeed, maintaining enzyme reserves of the glyoxylate shunt pathway by pre‐expressing aceBA prior to glucose runout reduces the lag‐time (Fig EV1C and D). Yet, why does it take so long for the few required enzyme types to reach sufficient concentrations for growth to resume?

To tackle this question, we next followed gene expression during the course of the shift. Translation rates are known to severely fall with growth arrest upon glucose depletion (Madar & Zaritsky, 1983; Erickson *et al*, 2017). Given these low rates, the high stability of proteins synthesized before the glucose runout, and the technical challenges to detect low levels of novel proteins, it is difficult to analyze the proteome response in high resolution. In contrast, given the fast turnover of mRNA (Chen *et al*, 2015; preprint: Balakrishnan *et al*, 2021), transcriptomics and the pool of mRNA species provide a good readout of momentary gene expression during the shift. Using RNA sequencing (RNA‐Seq), we determined mRNA abundances at six different time points. The mRNAs of the glyoxylate shunt and gluconeogenesis genes, represented as fraction of total mRNA, increase immediately (< 5 min) following glucose depletion, and these increased levels are maintained through the duration of the growth lag (Fig 1C). Given such a rapid regulatory response, the speed at which the transcriptional program changes is likely not the reason for long lag‐times, but it is rather the expression strength that could be important. To test this idea, we first employed a strain in which the native promoter of the *aceBAK* operon is replaced by the titratable promoter P*_tet_* (Basan *et al*, 2020). In this strain, as increasing concentrations of the inducer chlortetracycline (cTc) are added at the moment of glucose depletion, growth recovery is progressively faster, from no recovery for over 10 h in the absence of induction to ~3‐h recovery at the highest cTc concentration used (Fig 1D). Since the *aceBA* expression levels prior to glucose depletion are unperturbed, and thus uniform among the cultures, the decrease in lag‐times with increasing cTc concentrations highlights the significance of the active response to changing conditions in determining the transition kinetics. Following these results, we wondered whether lag‐times emerge due to a fundamental competition for shared resources such as RNA polymerase and ribosomal activity, which could be particularly limited during the shift: If a larger portion of the limited transcriptional and translational fluxes are allocated to the synthesis of the required mRNAs and proteins (Fig 1E top), the shunt and gluconeogenesis enzymes become available to replenish precursors earlier than in the case with a lower allocation of resources toward these genes (Fig 1E bottom).

**Figure 2:**
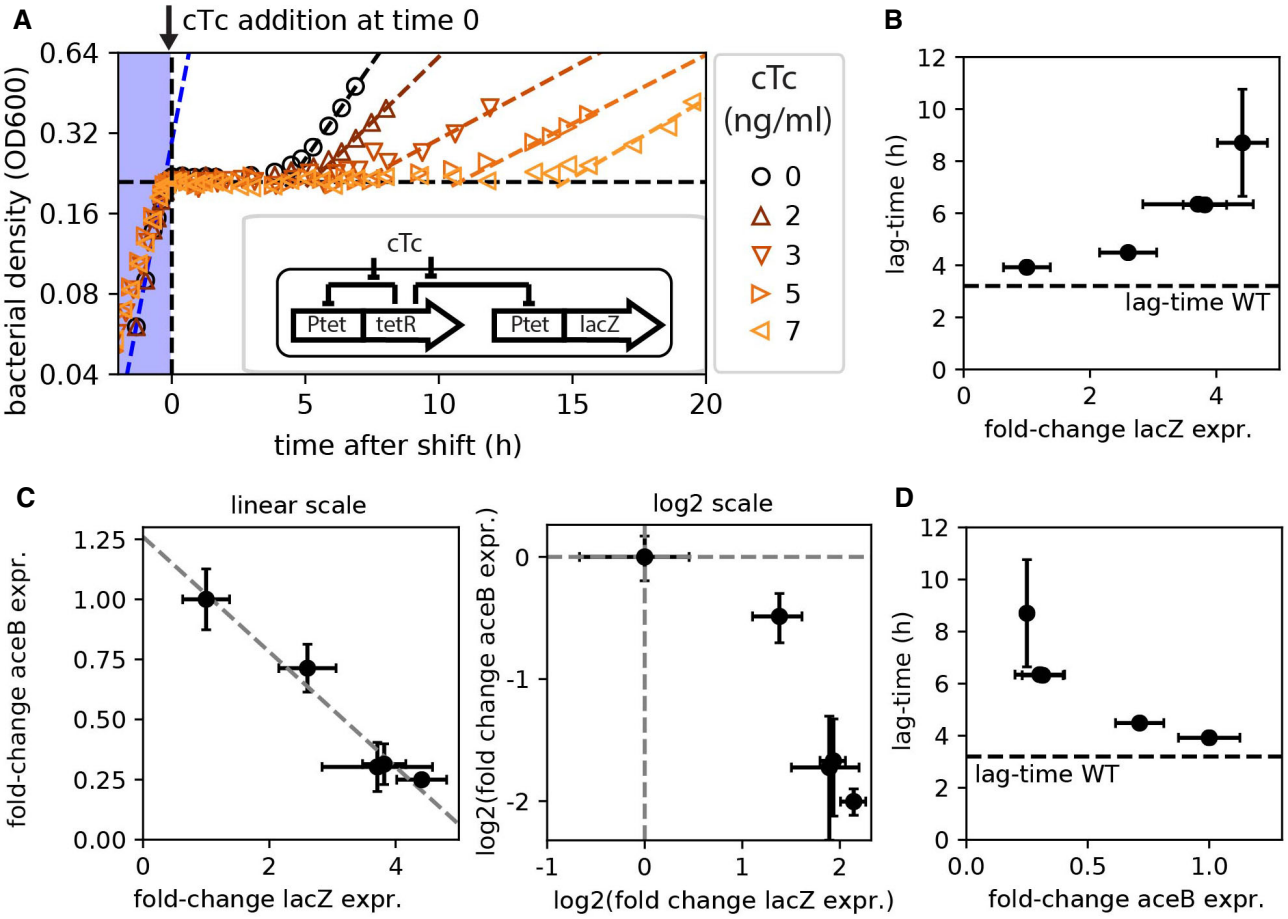
Expression of a non‐needed gene inhibits expression of required genes and elongates lag‐times AA plasmid system (inset) is used to control the expression of the non‐required gene *lacZ* using the strain NQ1389. *lacZ* expression was induced using cTc to varying degrees at the moment of glucose depletion (time = 0 h) using the indicated range of cTc concentrations. Diauxic growth conditions with glucose and acetate, same as in Fig 1.B–D
*lacZ* mRNA resulting from the different degrees of induction and *aceB* mRNA in the same cultures were measured by qPCR and plotted as fold change increase compared with that in the absence of induction. The lag‐times observed in panel A are plotted against the respective change in *lacZ* abundances (B), where the dashed line represents the 3.5‐h lag observed for the WT strain (no induction, Fig 1A). Changes in *lacZ* and *aceB* mRNA levels are inversely related (C), shown both as linear (left plot) and as log_2_ (right plot) scales. The dashed line in the left plot shows a linear fit. *aceB* mRNA abundance is inversely related to the lag‐time (D). Means of *N* = 3 and *N* = 5 biological replicates are shown for lag‐times and expression levels, respectively, in panels B–D. Error bars denote SD. Source data are available online for this figure.

**Figure EV2:**
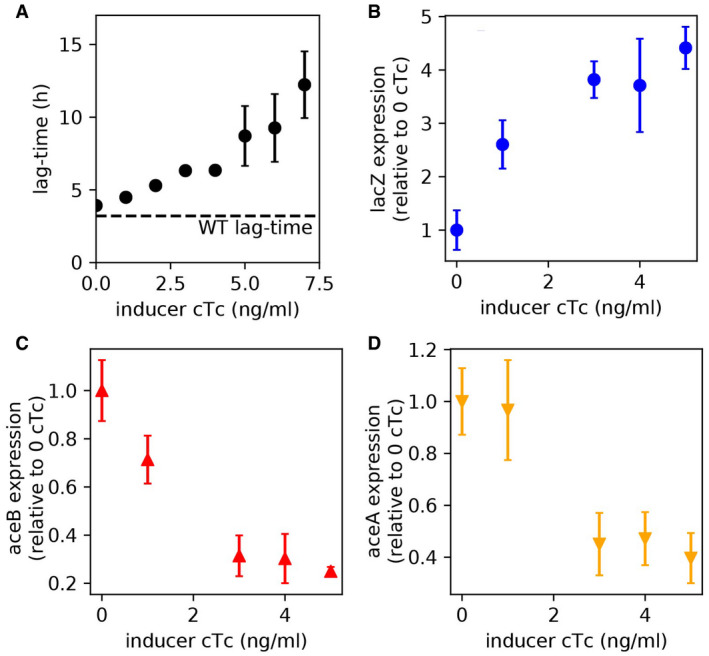
Lag‐times and gene expression when overexpressing lacZ AIncrease in lag‐times with increasing inducer levels (strain NQ1389). The inducer chlortetracycline (cTc) is added when glucose runs out (Fig 2A, time = 0). Dashed horizontal line indicates lag‐time for the WT strain (NCM3722).B–DmRNA levels of *lacZ*, *aceB*, and *aceA* at different cTc levels are quantified by qPCR 10 min after the depletion of glucose. mRNA levels of each gene are normalized to the 16S rRNA level, which is known to remain constant during the lag phase (Bosdriesz *et al*, 2015; Erickson *et al*, 2017). These normalized expression levels are shown relative to the expression level in the absence of induction (0 cTc). Data information: Mean of *N* = 3–5 biological repeats shown. Error bars indicate SD. Source data are available online for this figure.

To probe this allocation picture, we next employed a titration construct to overexpress *lacZ* (Scott *et al*, 2010), the product of which hydrolyses lactose and is thus useless for growth in glucose and acetate (Fig 2A, inset): When transcriptional and translational resources are diverted toward LacZ synthesis during the response to changing conditions, the protein itself adds no benefit to the cell and thus acts as a sink for shared resources, which should extend lag‐times. In line with this expectation, when inducing *lacZ* expression by adding various levels of the inducer chlortetracycline (cTc) at the moment of glucose depletion, we observed that lag‐times increase strongly from *τ_lag_* = 3.9 h at 0 ng/ml cTc to *τ_lag_* = 12.2 h at 7 ng/ml cTc (Figs 2A and EV2A). To further explore this effect, we measured the mRNA levels of *lacZ* and the required shunt genes *aceB* and *aceA* by qPCR, 10 min after the shift. The abundance of *lacZ* mRNA increases with inducer concentration (Fig EV2B) in direct relation to the lag‐time (Fig 2B). Notably, as *lacZ* mRNA is dialed up, *aceB* and *aceA* expression is reduced (Figs 2C and EV2C and D), explaining the longer lag‐times based on a lower expression of these required enzymes (Fig 2D). Hence, upon synthetically introducing a resource scarcity during an environmental shift, these observations indeed suggest that the allocation of limited shared resources determines the cellular response and thus lag‐times.

**Figure EV3:**
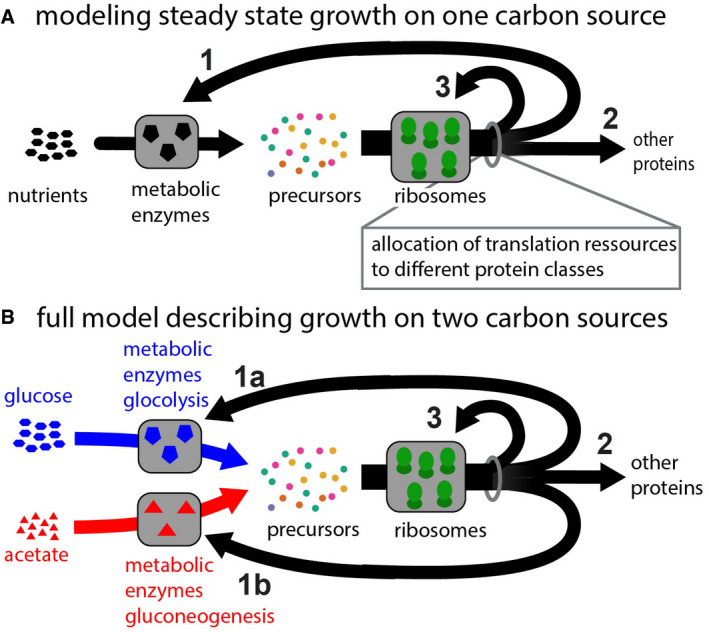
Modeling growth in changing environments A, BTo model growth during transitions, we here build on replicator/allocation models, which have been established previously for growth in steady‐state conditions and for specific shifts. Replicator/allocation models consider protein synthesis by ribosomes toward different proteins (Scott *et al*, 2014), and central to their approach is the allocation of ribosome activity toward the synthesis of different proteins. To illustrate the concept, we here consider steady growth on one carbon source first (A). The pool of ribosomes is allocated toward different protein classes such as the proteins for novel ribosomes (A, arrow 3), metabolic proteins needed to provide the precursors when utilizing the carbon source (A, arrow 1) and other proteins (A, arrow 2). Growth depends on the availability of nutrients and how the ribosomal activity is allocated to the different protein classes: High growth rates are achieved with allocation ratios that balance precursor influx provided by the metabolic enzymes and their utilization by ribosomes, such that as many ribosomes as possible can translate at maximum speed. This logic is formulated mathematically in Materials and Methods, and Appendix Supplementary Text. Notably, for *Escherichia coli* the allocation of ribosomal activity toward the synthesis of new ribosomes follows indeed a close to optimal regulation scheme preventing the synthesis of idle ribosomes (as manifested in the ribosome content changing with growth rate). To describe growth during shifts, we extend this modeling approach and explicitly consider glucose and acetate as two nutrient sources (B). In this case, two metabolic protein classes (arrows 1a and 1b), which provide the precursors when cells grow on the two carbon sources (blue and red arrows) and perform glycolysis (blue) or gluconeogenesis (red), respectively. As such, this model structure shares similarities with recent modeling approaches to describe growth during nutrient shifts (Molenaar *et al*, 2009; Erickson *et al*, 2017; Korem Kohanim *et al*, 2018). However, our approach is distinguished from those studies by the inclusion of two key aspects, which are central to the cellular response during growth shifts: (i) We consider the highly responsive regulation of transcription and integrate our transcription measurements (Fig EV5), which quantifies the immediate expression response of the cell during the shift. (ii) We explicitly vary the allocation toward other proteins shift (arrow 2) during the shift, thereby bringing the focus of the study to the allocational constraints acting during the response itself. Notably, right after the shift the model simplifies to the scenario shown in Fig 3A and the consideration of metabolic enzymes (arrows 1 and 2) together with the precursors required to drive novel protein synthesis is sufficient to investigate how long lag‐times can emerge and how they relate to the expression of non‐required proteins. The mathematical formulation of the full model and additional context is provided in Materials and Methods, and results for the switch from growth on glucose to growth on acetate are provided in Fig EV4. The central model output, the curve describing how lag‐times decrease with an increasing allocation to required proteins, is shown in Fig 3B.

**Figure 3:**
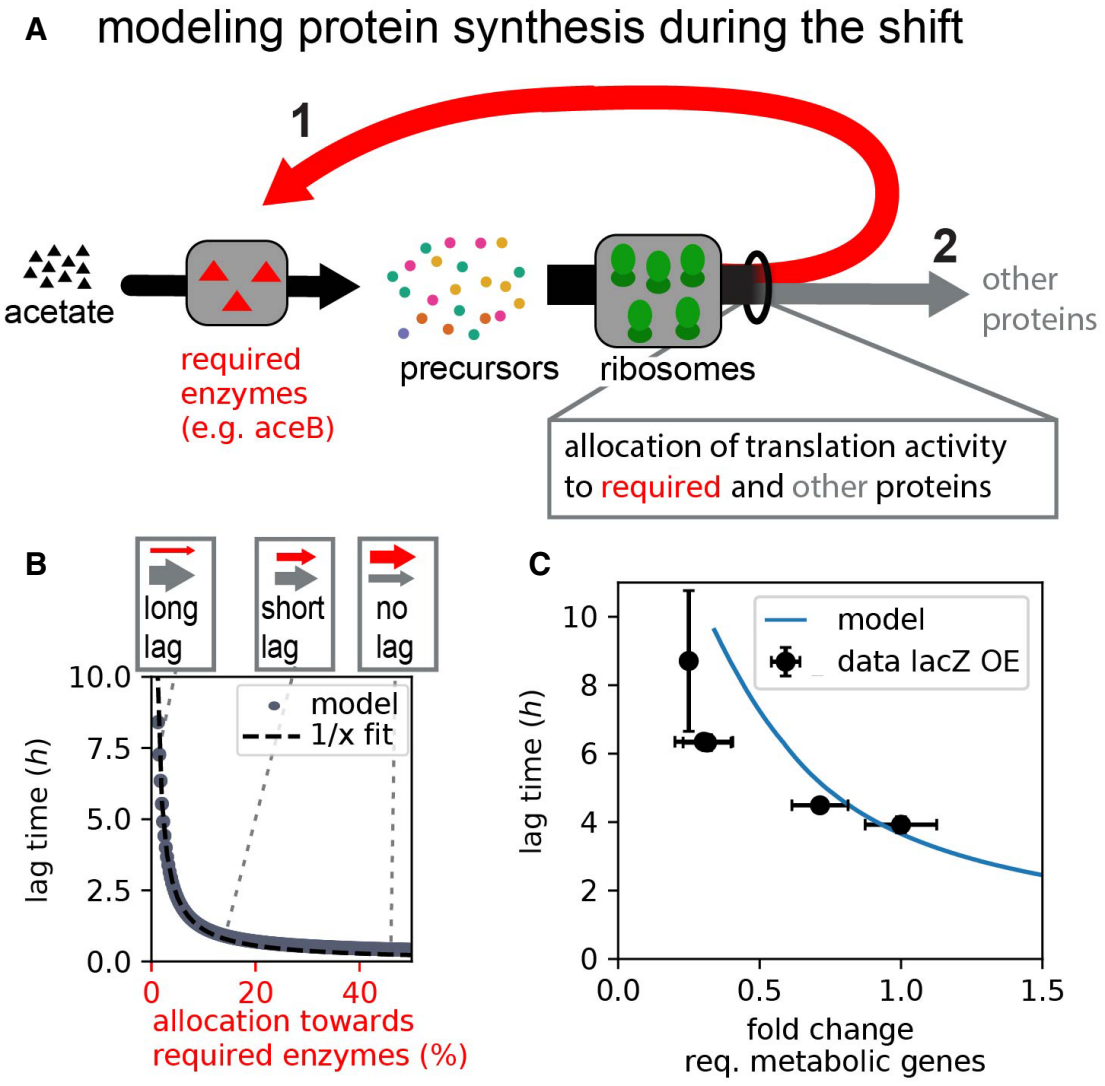
Modeling growth kinetics during the shift Essential dynamics during the shift from growth on glucose to growth on acetate: Protein synthesis by ribosomes depends on the availability of biosynthetic precursors, which itself depends on the availability of metabolic enzymes that utilize acetate to provide novel precursors. When ribosomes synthesize more of these required enzymes (red arrow 1) instead of other proteins (gray arrow 2), novel precursors are generated from acetate (black arrow) faster, and growth thus resumes faster (detailed considerations and full model introduction in Figs EV3 and EV4 and Materials and Methods).Lag‐times fall reciprocally with the allocation toward required proteins (the fraction of mRNA encoding for required proteins) during the shift. Allocation of translation activity toward different proteins is represented by the weight of red and gray arrows (top).Comparison of model prediction with observed changes in lag‐time when overexpressing the non‐required gene lacZ (Fig 2). The model has one major free parameter, the metabolic rate describing precursor influx. We determined this parameter by comparing predicted and observed lag‐times in the absence of induction (Materials and Methods). The model then predicts the change in lag‐time without further fitting when lacZ expression was induced and the fraction of required genes fell as a consequence. Data and error bars represent mean ± SD of *N* = 3–5 biological replicates, as in Fig 2D. All model parameters are provided in Appendix Table S3. Results and figures can be regenerated using the Jupyter Notebook available on GitHub.

**Figure EV4:**
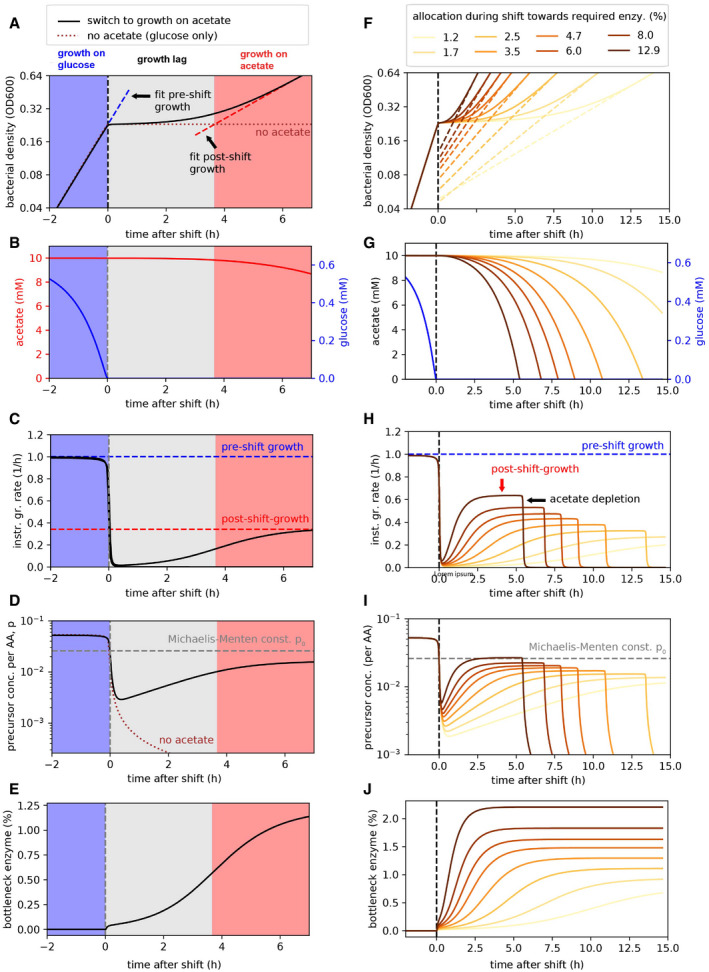
Modeling growth transitions and the consequence of non‐required protein expression Based on our model of growth transitions (Fig EV3, Materials and Methods), we analyzed growth kinetics during the transition from growth on glucose to growth on acetate. A–EChange of major model variables (cell density, nutrients, precursors, and required metabolic enzymes) during the shift for a reference condition with a specific allocation toward the metabolic enzymes required for growth on acetate. Growth and nutrient abundance: When glucose is available, cells grow fast (A, blue region) consuming only glucose, and not acetate (B, blue region). Following glucose depletion, precursors fall dramatically (D, gray region) and growth thus stops (A and C, gray region). Cells then only slowly synthesize the required metabolic enzymes such as AceB (E), which then slowly support higher precursor concentrations (D, gray region). The case where no metabolic proteins for growth on acetate are synthesized is shown for comparison (D, dashed line); precursor concentrations continue to fall. Growth only recovers to post‐shift growth rates (A and C, red regions) once precursors reach concentrations comparable again to the Michaelis–Menten constant (D, dashed horizontal line), which describes the concentration of precursors above which ribosomes can work efficiently with close to maximum translation rates. The growth recovery is slow and spans several hours since cells are trapped in a state where precursor concentrations and the abundance of the metabolic enzymes that can generate new precursors are both low. Accordingly, novel metabolic proteins can only be generated slowly and precursor concentrations thus also recover slowly.F–JEffect on changing allocation toward the metabolic enzymes required for growth on acetate. Plots show the same variables as in (A–E) but for different allocational behavior toward the synthesis of novel proteins required during the shift (model parameter $\alpha_{Mb,ace,max}$ describing the allocation of overall transcription (mRNA fraction) to the required enzymes; see color legend). A higher allocation toward the metabolic enzymes (darker colors) substantially decreases the lag during the growth transition as it leads to much faster accumulation of required metabolic enzymes (J), preventing a dramatic decrease in precursor concentrations right after the shift (I). This thus also leads to a much faster recovery of growth (I). Growth stops once acetate is also consumed (G, H). The change in lag‐times for different allocations toward the required metabolic enzymes is shown in Fig 3B. Data information: All times shown are relative to the time point where glucose is depleted, and colored regions in (A–E) indicate different growth phases as defined by the intersection of exponential growth curves (dashed lines in A and F) and the density values during the shift, following what was done for the experiments (Figs 1A and EV1A). Model parameters as listed in Appendix Table S3.

To better understand how the competition for shared transcription and translation resources can have such drastic impacts on growth transitions, we next formulated a kinetic model of growth, which focuses on protein synthesis as the most resource demanding process of biomass synthesis (detailed description in Materials and Methods). The model builds on recent advances to describe growth (Molenaar *et al*, 2009; Scott *et al*, 2014; Hermsen *et al*, 2015; Erickson *et al*, 2017; Allen & Waclaw, 2018; Korem Kohanim *et al*, 2018) and explicitly considers amino acid precursors, their synthesis by metabolic enzymes, and their utilization by ribosomes in form of charged tRNA (Fig EV3). A key feature of the model is that only a fraction of the ribosomes synthesizes the enzymes (e.g., *AceB*) that supply the precursors, while the rest of the translation flux is diverted to the synthesis of other proteins (Fig 3A). The consumption of amino acid precursors, however, depends on the (total) protein synthesis, leading to a feedback between protein synthesis and precursor supply. During the diauxic transition, where there is a sudden depletion of cellular amino acid pools following the runout of the preferred carbon source, this can lead to cells being “trapped” in a low precursor state. The mathematical analysis shows that such states can persist for hours when (i) the required proteins such as AceB have not been synthesized in sufficient numbers yet, and (ii) the remaining amino acid levels are insufficient to support the synthesis of new proteins (Fig EV4A–E). A direct way to mitigate this trap is to allocate a larger fraction of the translation flux toward the synthesis of the required enzymes (Fig EV4F–J). Accordingly, lag‐times fall drastically with a higher allocation toward the synthesis of required enzymes (Fig 3B), reflecting the lag‐time changes observed when overexpressing the required or non‐required genes *aceBA* and *lacZ* (Figs 1D and 2). A quantitative comparison between the model prediction and the observed lag‐times upon non‐required gene (lacZ) expression is shown in Fig 3C. Taken together, our experiments and theoretical analyses establish mechanistically how the allocation of limited resources during the shift can shape growth transitions, outlining a range of possible allocational behaviors with varying consequences on the growth transition kinetics.

**Figure 4:**
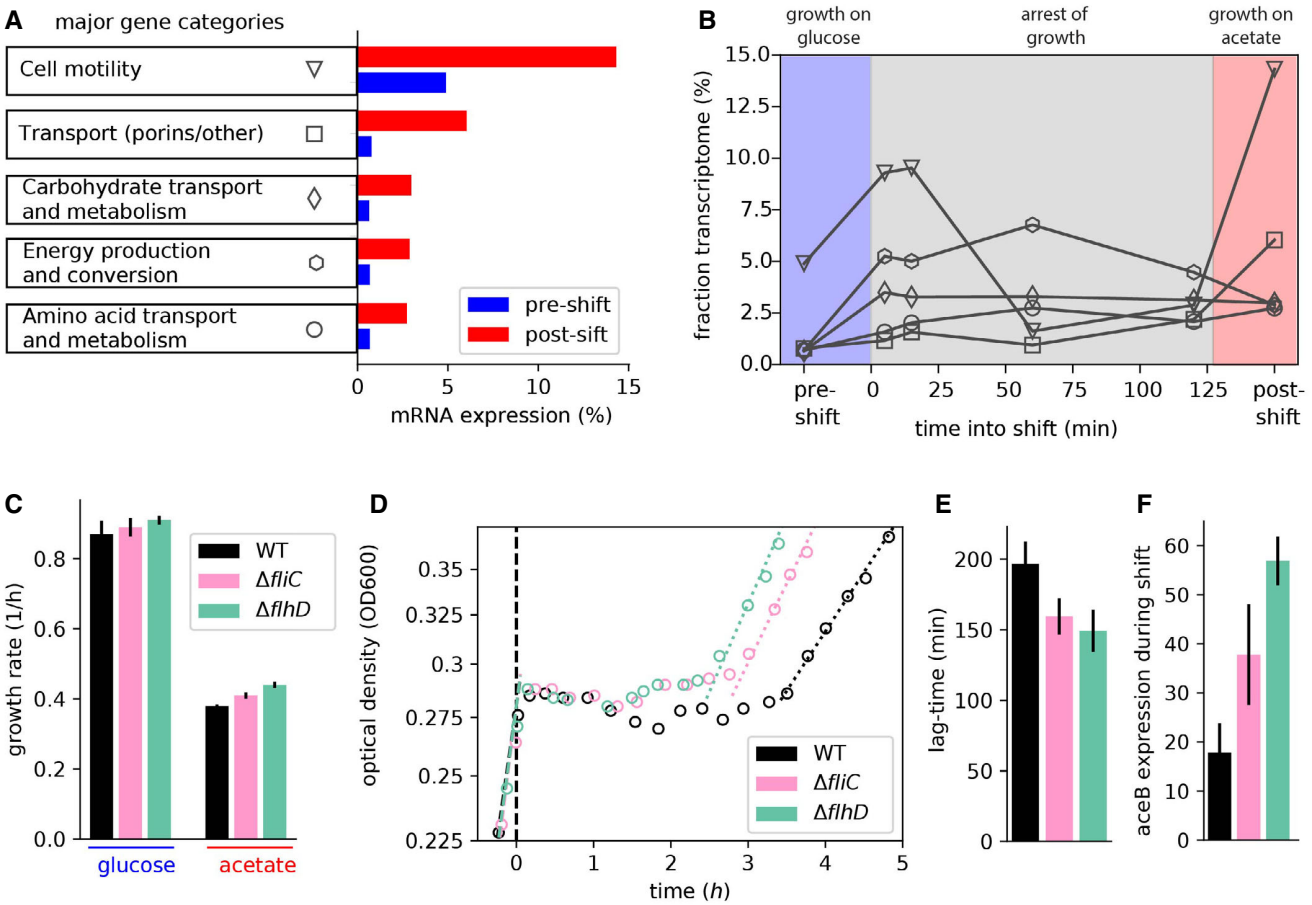
Expression of non‐required genes that delay growth recovery during the glucose acetate shift AThe major gene categories upregulated in balanced growth on acetate (red) compared with that on glucose (blue). mRNA abundances determined by RNA‐Seq are represented as percent of total mRNA in the given growth condition. Genes are categorized using the COG classification (Tatusov *et al*, 2000), and porins were classified by manual curation using annotations available from ecocyc.org.BFor the various gene categories in panel A, the temporal kinetics of expression during the diauxic shift is plotted. Time 0 indicates time when glucose runs out. The series of RNA‐Seq through the growth transition was performed once.CSteady‐state growth in glucose and acetate for the WT and the motility deletion strains *ΔfliC* and *Δflh*. Growth rate differences between WT and the mutants are not significant in glucose (*P*‐value > 0.05), but are significant for growth in acetate (*P*‐values < 0.02). Additional growth conditions are shown in Fig EV6.D, EGrowth transitions for the motility deletion strains *ΔfliC* and *ΔflhD* are substantially faster than that for the WT strain, with (D) showing representative growth kinetics of the three strains and (E) showing mean lag‐times and standard error of four independent biological replicates. Lag‐times for WT are significantly different from that of *∆fliC* (*P*‐value 0.01) and *∆flhD* (*P*‐value 0.004).F
*∆fliC* shows a twofold increase (*P*‐value 0.17), and *∆flhD* shows a threefold increase (*P*‐value 0.02) in aceB expression compared with WT, as measured by qPCR. Data information: Mean of *N* = (4, 4, 2) biological repeats is shown in (C, E, F), respectively, with error bars denoting SD. All indicated *P*‐values were computed by a two‐sample *t*‐test. Source data are available online for this figure.

**Figure EV5:**
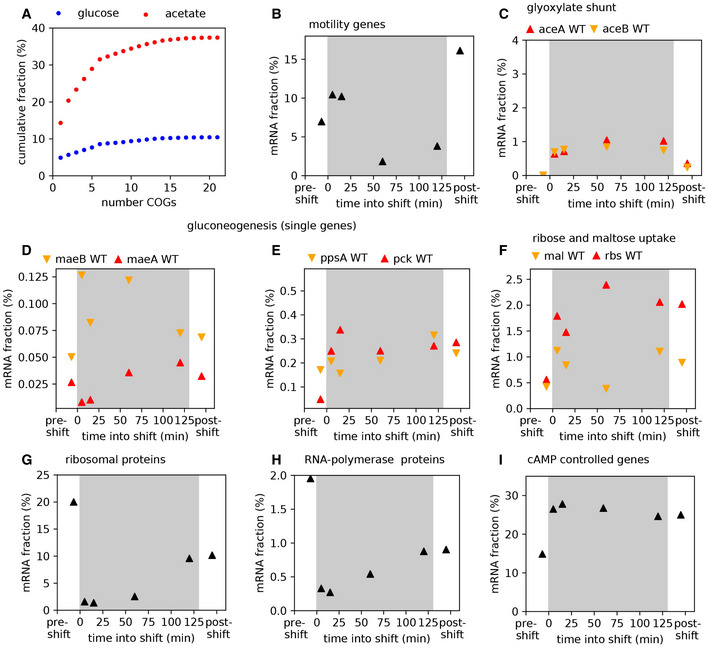
Gene expression during the transition AFractions of genes upregulated in acetate (red) compared with glucose (blue), shown as cumulative sum over most expressed functional groups. Data are derived from transcriptomics measurements (RNA‐Seq) using adjusted clusters of orthologous groups (COGs). The five most expressed functional groups, shown in Fig 4B during the shift, cover most of the genes that are upregulated.B–ImRNA abundance for different genes and gene groups during the diauxic shift from glucose (pre‐shift) to acetate (post‐shift), using the RNA‐Seq measurements. (B) Motility genes (defined in (10), consisting of *fliC* encoding the major structural component of the flagella and different motor genes) are heavily expressed during the shift. (C) Glyoxylate shunt genes (detailed version of Fig 5D) are upregulated. (D, E) The upregulation of different gluconeogenesis genes. (F) *rbs* and *mal* genes (detailed version of Fig 5C) are upregulated during the shift. (G, H) Expression of ribosomal protein (summed over all *rpl*, *rpf*, and *rpm* genes) and RNA polymerase genes (*rpoA,B,C*) during the shift. Within 5 min, these genes are repressed, confirming a responsive downregulation mechanism. (I) The expression of genes known to be under the control of the second messenger cAMP (Kalisky *et ai*, 2007). This group of genes is upregulated during the shift. All data are derived from the same RNA‐Seq dataset taking the relative fraction of different genes (and their sum when groups of genes are considered). Series of measurements during the shift was performed once and with the WT strain NCM3722. Source data are available online for this figure.

**Figure EV6:**
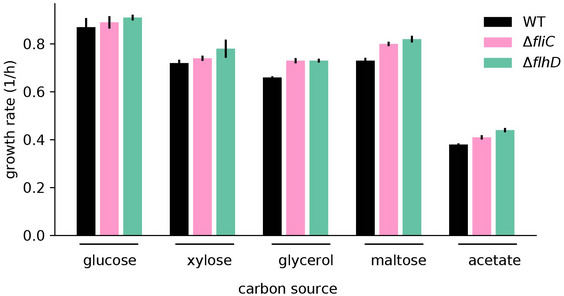
Balanced growth of deletion strains Steady growth of WT and motility deletion strains *ΔfliC* and *ΔflhD* (strains listed in Appendix Table S2). Growth with 20 mM glucose, 20 mM xylose, 20 mM glycerol, 20 mM maltose, and 30 mM acetate. Data for growth on glucose and acetate as shown in Fig 4C. Mean of *N* = 4 biological repeats are shown. Error bars indicate SD. Differences in steady‐state growth rate between WT and the mutants are significant in each case besides glucose (*P*‐value < 0.05, except for ∆fliC in xylose which has a *P*‐value of 0.07). All indicated *P*‐values were computed by a two‐sample *t*‐test. Source data are available online for this figure.

We next ask where in this range of allocational behaviors does native *E. coli* (no synthetic overexpression) fall, and whether the long lag‐times observed for WT *E. coli* (Fig 1A) also emerge from the synthesis of non‐required proteins during the shift. It has long been known that *E. coli* growing steadily on poor carbon sources (such as acetate and glycerol) express several catabolic enzymes, despite the absence of their specific substrates (Hui *et al*, 2015; Schmidt *et al*, 2015). Allocating resources toward such “idling” proteins during growth transitions would map *E. coli* toward the left of the plot in Fig 3B, with lag‐times substantially larger than those expected with specialized regulatory strategies in which only the required genes are expressed. To estimate the transcriptome fraction that potentially encodes idling proteins, we first analyzed transcriptomics measurements for *E. coli* grown under steady‐state conditions with either glucose plus acetate (representing the pre‐shift transcriptome) or only acetate (representing the post‐shift transcriptome) as carbon sources. We considered the more abundant half of the genes and determined those genes that are expressed at least twofold more in acetate than in glucose plus acetate. Most of these genes belong to a few functional categories including transport, motility, and catabolism (Figs 4A and EV5A). Furthermore, as seen from our transcriptomics data collected over the course of the growth transition, these genes are upregulated immediately following glucose depletion (Fig 4B). Yet, most of these functions are not expected to be useful for the growth transition to acetate: Motility is not needed in shaking environments and most of the uptake transporters are involved in transporting other nutrients besides acetate. The expression of these non‐required genes could impede the allocation of shared resources toward the genes encoding for shunt and gluconeogenesis enzymes. To test this idea, we next considered the growth behavior of deletion strains lacking motility genes, the category which showed most increased expression in acetate (Fig 4A). We considered two mutants, *ΔfliC* and *ΔflhD*, which either do not express the flagella protein FliC or the motility master regulator FlhDC required for the expression of flagella, motors, and the chemotaxis apparatus (preprint: Honda *et al,* 2021). The mRNAs of these genes comprise up to ~15% of the total transcription in the WT strain growing on acetate and could considerably reduce the allocation of resources toward the shunt and gluconeogenesis genes. When grown under steady‐state conditions with either glucose or acetate as sole carbon sources, we observe that none of these deletion strains have any defect in steady growth rates, but rates even increased substantially for growth on acetate (Fig 4C), supporting the idea that these gene products are indeed useless for growth in the probed conditions. Notably, the increase in growth was also observed for other carbon sources besides acetate (Fig EV6) and provides direct support for the idea that gene regulation is not optimized for steady‐state growth (Ibarra *et al*, 2002; O'Brien *et al*, 2016; Towbin *et al*, 2017). These results corroborate similar conclusions drawn in other bacteria, including studies demonstrating the proteome burden of expressing the light‐harvesting machinery in the cyanobacteria *Synechocystis* (Jahn *et al*, 2018), and of motility genes in *Pseudomonas putida* (Martínez‐García & de Lorenzo, 2011).

Given the vast expression of motility genes immediately following glucose depletion (Fig 4B), we next probed how the synthesis of these non‐required proteins affects the growth transition from glucose to acetate by quantifying lag‐times for the two deletion strains. Lag‐times are shorter for the deletion strains than for the WT strain (Fig 4D and E), suggesting that the deletion strains are better at directing RNA polymerases and ribosomes toward the synthesis of the shunt and gluconeogenesis enzymes. To test whether the deletions lead to increased transcription of the required genes, we used qPCR to track the upregulation of the glyoxylate gene *aceB* during the transition. The *ΔfliC* and *ΔflhD* strains indeed show an up to threefold higher upregulation of *aceB* compared with the WT (Fig 4F), explaining the reduced lag‐times based on the higher resource allocation toward the required genes. These results are consistent with the previous observation that the overexpression of non‐needed genes leads to lower *aceB* expression levels and thus longer lag‐times (Fig 1).

**Figure 5:**
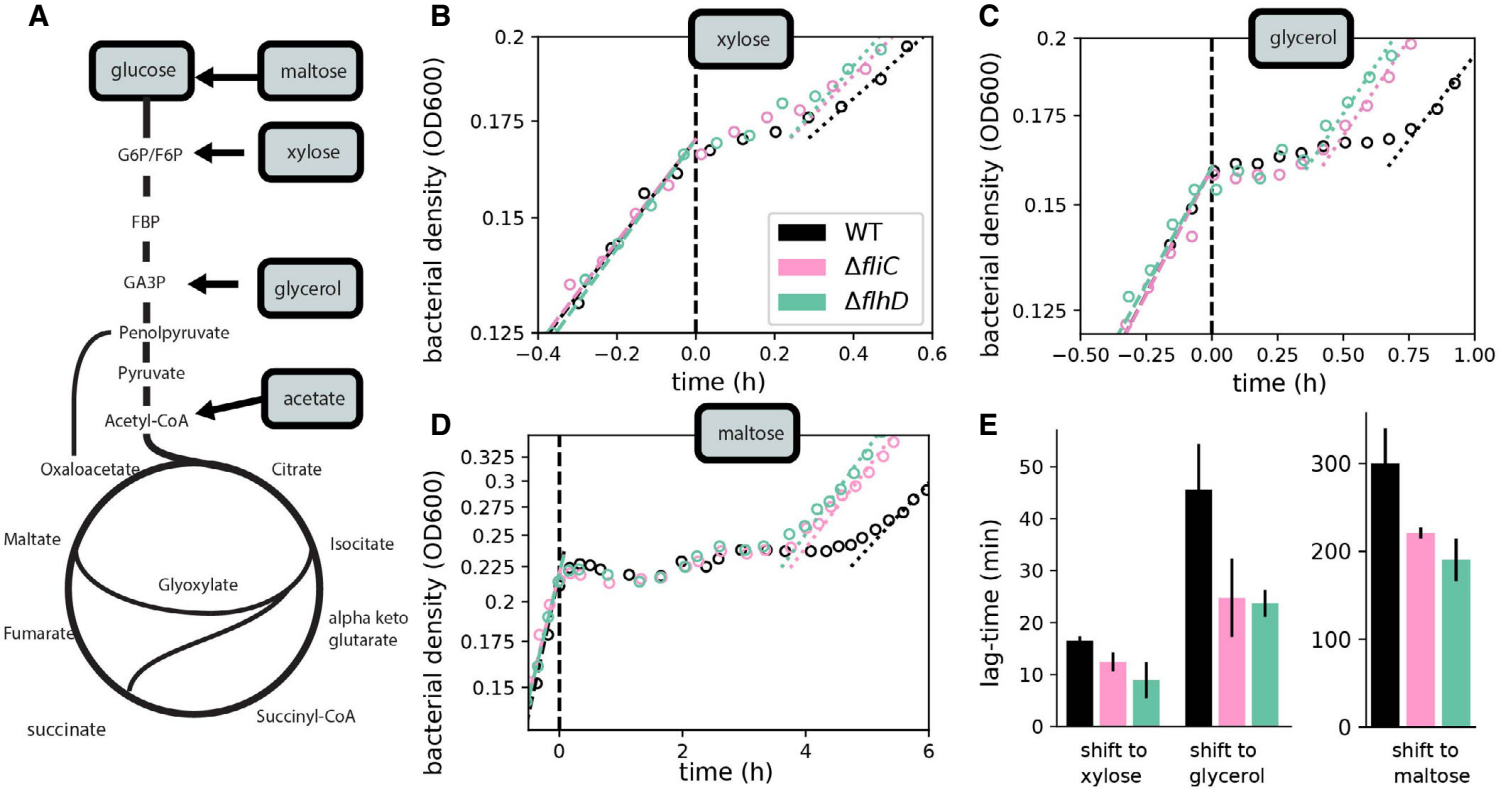
Expression of non‐required genes is responsible for lags during many diauxic shifts AThe expression of different uptake and metabolic proteins is required for the utilization of different carbon sources. Points of entry into the central metabolism are indicated.B–DDiauxic growth kinetics for growth on glucose and a different secondary carbon sources for the WT (black) and the motility deletion strains *ΔfliC* and *ΔflhD* (magenta and green).EDerived lag‐times for the different growth transitions. Means based on *N* = 4 biological repeats are shown. Error bars denote SD, and lag‐time difference between WT and the two mutants is significant for shift to each transition (*P*‐value < 0.05; two‐sample *t*‐test). Source data are available online for this figure.

Finally, given the results for the diauxic growth on glucose and acetate, we asked whether the expression of idle proteins and their competition with required metabolic enzymes is also responsible for the lag‐times observed in other diauxic growth conditions. We tested other conditions originally reported by Monod (1949, 1966), inducing shifts from glucose to glycerol, xylose, and maltose. These carbon sources enter the central metabolic pathway at different steps (Fig 5A) and do not require the flux reversal from glycolysis to gluconeogenesis, which was recently suggested to explain long lags in growth transitions (Loomis *et al*, 1967). Instead, these carbon sources necessitate the synthesis of other unique sets of transporters and catabolic enzymes for growth to resume and are thus distinct from acetate. Using the motility deletion strain, we consistently find reduced lag‐times for all transitions (Fig 5B–E). Hence, the allocational constraint of shared protein synthesis resources is a general principle governing a range of different diauxic transitions.

## Discussion

Since the pioneering growth physiology studies by Monod, lag‐times have been perceived as the preparation time cells require to adjust to a new environment before growth can resume (Monod, 1949, 1966; Stanier, 1951; Epstein *et al*, 1966; Fernández‐Coll & Cashel, 2018; Bertrand, 2019). In line with this idea, several experimental studies in bacteria and yeast suggest that the expression of required genes before the nutrient shift can reduce lag‐times but can come with the cost of slower growth rates, implicating a trade‐off between lag‐times and growth in the pre‐shift condition (Siegal, 2015; Venturelli *et al*, 2015; Wang *et al*, 2015; Chu & Barnes, 2016; Basan *et al*, 2020). Theoretical studies have further rationalized some of the observed growth kinetics assuming cells optimize protein utilization and metabolic fluxes in encountered conditions (Beg *et al*, 2007; Kalisky *et al*, 2007; Wang *et al*, 2019).

In this quantitative study, we instead focus on the cellular response to environmental changes and find that the response is not optimal in the conditions probed: We establish how the competition for shared resources toward novel protein synthesis fundamentally constrains the cellular response. The duration of growth recovery depends on whether only the required genes (specific response) or a diverse array of genes (diversifying response) are expressed as response to the environmental change. Tweaking the specificity of the response can substantially vary lag‐times, and WT *E. coli* express a diverse array of genes leading to long lag‐times.

Our observations thus call for a revision of the previously proposed explanations for growth transitions: While pre‐shift growth/lag trade‐offs may affect lag‐times, growth transitions are determined first and foremost by a compromise between specific versus diversifying responses during the shift. Mechanistically, this compromise stems from a constraint on the allocation of shared resources toward the synthesis of novel proteins during the response. This constraint opens a multitude of possible response strategies, ranging between extremely specific and diversifying mechanisms. A highly specific response tailored toward the growth conditions encountered can ensure fast growth transitions across many shifting conditions. But harboring specific regulatory pathways for multiple different nutrient sources can be cumbersome. Indeed, cost of regulation has been suggested to play a role in shaping regulatory strategies (Kalisky *et al*, 2007). In addition, a diversifying response involving the expression of a broad array of genes might provide benefits in certain ecological scenarios.

A series of recent studies have explained certain diauxic transitions in bacteria and yeast on the basis of population heterogeneity (Kussell & Leibler, 2005; Kotte *et al*, 2014; Solopova *et al*, 2014; Grimbergen *et al*, 2015). Populations may “bet‐hedge” through variable gene expression among individual cells to maximize the chances of successfully coping with an environmental change. The proteome allocation constraint during cell response as is revealed in this study is a potential driving factor underlying such bet‐hedging strategies that are enforced through population heterogeneity. It would thus be interesting to explicitly investigate how the allocation constraints shape bet‐hedging strategies. For the diauxic condition probed in this study, however, no heterogeneity was observed (Basan *et al*, 2020). Cells thus also employ deterministic gene regulatory circuits to homogenously respond to encountered conditions.

The response of *E. coli* includes the expression of several genes such as diverse transporters and flagella components, which may not be required in the encountered environment. The response is thus diversifying instead of specific, which may be rationalized from an ecological perspective: Besides glucose, *E. coli* encounters many other sugars and amino acids within the mammalian intestine, and swimming is expected to play a crucial role in the strong flow environment prevalent within the intestine (Cremer *et al*, 2016, 2017). The diversifying response thus appears to be tailored toward coping with the fluctuating environments typical for the gut. But it is exactly such a response that constrains resource allocation toward the specific required genes during other transitions, as those encountered in laboratory experiments, leading to long lag‐times. Accordingly, strains evolving in typical laboratory environments would be expected to lose their diversifying response. In fact, we observe evidence for this hypothesis embedded in previously published results of the long‐term evolution experiment by Lenski & Travisano (1994) (Good *et al*, 2017): The evolved strains exhibit loss of growth on many carbon sources and motility (Leiby & Marx, 2014). In light of our findings, it would be interesting to see whether the selective advantage for these evolved strains is indeed, in part, due to shorter lag‐times. Finally, by describing the mechanistic constraints shaping the cellular response, our work establishes the physiological framework for rational strain engineering in biotechnological applications: By trimming down the diverse response, highly optimized behaviors such as short lags and high yields can be instilled.

## Materials and Methods

### Strain information

The wild‐type strain we use is the extensively characterized *E. coli* K‐12 strain NCM3722 (Soupene *et al*, 2003; Brown & Jun, 2015). This strain was also used as the parent for the construction of all the strains used in this study. All strains are listed in Appendix Table S1. To obtain the fliC and flhD deletion strains, the corresponding KO strain JW1908 from the Keio collection (Baba *et al*, 2006) was used and the deletion was subsequently moved into the NCM3722 strain by phage P1vir transduction, yielding strains GE029 and NQ1225. Used primers are listed in Appendix Table S2.

### Media and growth conditions

All growth media used in this study were based on the MOPS‐buffered minimal medium used by Cayley *et al* (1989) with slight modifications (Hui *et al*, 2015). 20 mM NH4Cl was provided as nitrogen source. One of the following substrates was used as the primary carbon source: 20 mM glucose, 30 mM acetate, 20 mM glycerol, 20 mM sorbitol, 20 mM succinate, 6 mM mannose, 4 mM mannose, and 20 mM xylose. For shift experiments, cells were grown with 0.61 mM glucose and the concentration of the second carbon source as stated before (e.g., 30 mM acetate). For the titratable strains (NQ1389 for lacZ and NQ1350 for aceBA), different concentrations of chlortetracycline (cTc) to induce expression and 15 μg/ml chloramphenicol and 50 μg/ml ampicillin to maintain the plasmid construct were additionally provided.

Cells were grown in a 37°C water bath shaker shaking at 250 rpm. To ensure balance growth, cells grew exponentially for at least seven generations before starting measurements. We measured optical density at 600 nm (OD600) using a UV‐Vis spec. To obtain the growth rate of steadily growing cultures, OD600 data points within the range 0.04–0.4 (linear range of spectrophotometer) were obtained and fitted to an exponential growth curve. In addition, growth curves and transitions were also quantified in a microplate reader (200 μl per well). A Tecan Spark Microplate Reader was used, and absorbance (420 nm) was measured every 7 min; incubation temperature was set to 37°C. Between measurements, plates were shaking at 132 rpm with an orbital amplitude. Wells loaded with only media (no culture) were used to reset absorbance values, and obtained absorbance values were subsequently adjusted to obtain OD600 values matching the values obtained with the UV‐Vis spectrophotometer and a path length of 1cm. Obtained growth rates for glass tube cultures and incubation in the microplate reader are highly comparable (< ± 5% difference). Growth rate measurements were repeated several times as indicated in the figure captions.

### Lag‐time quantification

To quantify lag‐times, we first fit exponential growth behavior to the two steady growth phases (growth on glucose and growth on acetate) using OD600 ranges (0.04…0.15) and (0.3…0.4) for growth on glucose and acetate, respectively. Plateau levels (no change in OD600) were then determined by hand (OD value that first derivatives from the exponential growth on glucose), and the times *t_pl,glucose_* and *t_pl,acetate_* where the exponential curves match the plateau levels were calculated. The lag‐time is the difference of these times, *t_lag_* = *t_pl,acetate_* − *t_pl,glucose_*. Times were readjusted before plotting (*t* → *t* − *t_pl,glucose_*, such that time=0 corresponds to the time where the exponential growth on glucose hits the plateau level (beginning of shift). The lag‐time estimation is further described in Fig EV1A. Experiments to quantify lag‐times were repeated at least three times as indicated in the figure captions.

### Overexpression experiments

Overnight pre‐cultures (OD600 ~0.5) were diluted to a starting OD600 of ~0.02. To ensure a simultaneous entry into the shift phase (time and density glucose runs out) for different inducer levels, a main culture was prepared in an Erlenmeyer flask, and after 1 h of incubation, this main culture was split into different cultures (glass tubes with 6 ml culture each). A different amount of inducer stock (cTc) was then added for the different cultures at the moment of glucose runout. To ensure the addition of the inducer exactly at this time, a control culturing starting with slightly higher cell density was run in addition, which entered the shift approximately 30 min earlier. The observed density value at the shift was used as the indicator when to add the inducer levels to the main cultures. Obtained lag‐times were highly reproducible for inducer levels up to 5 ng/ml. For higher inducer levels (lag‐times > 5 h), variation was higher, presumably because the slightly late addition of inducer levels leads to small variation in bottleneck enzymes, which can have strong consequences over long times.

### qPCR measurements

RNA was extracted using the TRIzol (Thermo Fisher) method combined with a column‐based purification step. In detail, 2 ml culture was collected 30 min after the shift (30 min after glucose runout) and spinned down. Pellet was immediately resuspended in 250 μl TMN buffer (10 mM Tris pH 8, 10 mM MgCl_2_, and 60 mM NH_4_Cl) and thoroughly mixed with 250 μl TRIzol. After 5‐min incubation, 50 μl chloroform was added, and after mixing and another minute incubation, the sample was centrifuged for 10 min at 4°C. The clear phase was collected, and the obtained RNA was immediately washed using a RNA purification kit (Zymo Research RNA Clean & Concentrator‐5, following the instructions). To remove plasmid DNA, a DNAse digestion step on the purification columns was added following the instructions. To quantify transcription, a two‐step qPCR approach was chosen. Reverse transcription was first run with random hexaprimers to obtain cDNA (Azura Genomics, AzuraFlex cDNA Synthesis Kit following the instructions with 1 µl of RNA sample). Real‐time PCRs (10 μl final volume) were prepared using a SYBR Green master mix (Bio‐Rad SsoAdvanced Universal SYBR Green Supermix), following the instructions and using 10× diluted cDNA sample. Primer sets for 16S RNA and the genes *aceB*, *aceA*, and *lacZ* were used as listed in Appendix Table S2. 300 nmol primer concentration was used, standard curves confirmed the linearity for each of the four primer sets chosen, and melting curves confirmed selectivity of the reaction. The PCR was run in a Bio‐Rad CFX 384 instrument, with the protocol following the reagent instructions provided with the master mix. To calculate relative expression levels, obtained gene expression levels were normalized to the 16S RNA levels measured from the same cDNA sample. The expression level of, for example, aceB was calculated as $2^{{‐(c}_{w,aceB}‐c_{W,16S})}$. To compare the changes in expression for different inducer levels, values were in addition normalized by the expression level obtained for 0 inducer level. Measurements were repeated at least three times starting with different cultures (biological repeats).

### RNA‐Seq sampling and analysis

Steady‐state cultures were grown to OD600 of 0.5. 10 ml samples were spun, and cells were resuspended in 200 μl TMN buffer (10 mM Tris pH 8, 10 mM MgCl_2_ and 60 mM NH_4_Cl). RNA was extracted using 200 μl TRIzol reagent followed by ethanol precipitation. Ribosomal RNA was removed using the Ribo‐Zero Kit (Illumina), and barcoded RNA‐Seq libraries were generated using the TruSeq stranded kits (Illumina) as per the vendor's protocol. The libraries were sequenced using Illumina's Hiseq 4000 platform. Typically, around 20 million reads were obtained per sample, except for the WT sample 5 min post‐shift, which had 5 million reads. Reads were demultiplexed and aligned to the *E. coli* MG1655 U00096.3 genome using bowtie v2.2.6 (Langmead & Salzberg, 2012). Read counts were obtained using Python HTSeq‐count (HTSeq v0.6.1p2) (Anders *et al*, 2015).

### Statistical analysis

Arithmetic means of growth rates, lag‐times, and qPCR measurements were based on *N* = 2–5 biological replicates as indicated in the respective figure captions. Error bars denote standard deviations. Statistical significance was probed by a two‐sample *t*‐test. The two‐tailed *P*‐values are listed in the captions and findings. Individual measurements, means, SD, and *P*‐values are provided in the source data file.

### Computational modeling

Formulating a model of growth transition: We introduce in this section in detail the allocation model to study growth transitions (model parameters are listed in Appendix Table S3, and the code used to solve the model is available via GitHub at https://github.com/jonascremer/lagtimemodeling). Modeling growth transitions is challenging because one needs to analyze how core growth processes change with current growth conditions, which depends on the metabolic state of the cell. More recently, several modeling approaches have been formulated to overcome these challenges and to investigate growth transitions in different changing environments, including defined down‐ and upshifts to carbon sources supplying faster or slower growth. These models consider the growth conditions during growth transitions focusing on the link to observed steady‐state growth behavior, parameterizing, for example, the fluxes such that they merge with steady‐state conditions (Erickson *et al*, 2017; Korem Kohanim *et al*, 2018). These approaches extended the logic of growth laws from steady‐state considerations to also describe the growth kinetics during the transition. To explicitly analyze the promoting effect of required metabolic proteins and the inhibiting effect of non‐required genes on growth transitions, we built on these models but chose a more explicit approach and specifically considered the expression of required and non‐required enzymes during the shift. Model logic and details are provided in the following. Model results are shown in Figs 3 and EV4.

Growth on one nutrient source: To model for growth kinetics, we first introduce a simpler model for the growth on one carbon source. The model focuses on novel protein synthesis, the most resource demanding cellular component, and considers the allocation of ribosome activity to different protein classes as introduced in Fig EV3. We specifically build on the rationale introduced in Scott *et al* (2014) to first consider balanced growth on one carbon source. Proteins are synthesized by ribosomes, and the increase in total protein mass in the culture (variable *M*) depends on the number of ribosomes *N_Rb_* in the culture and their average translation speed *k_Rb_* (how many new amino acids a ribosome is synthesizing per time):

$$\frac{\mathrm{dM}}{\mathrm{dt}}=\;k_{\mathrm{Rb}}N_{\mathrm{Rb}}.$$

For convenience, we measure protein mass in numbers of amino acids such that no extra conversion factor is needed. Instead of the number of ribosomes, we can also consider their mass (variable *M_R_*) writing:

$$\frac{\mathrm{dM}}{\mathrm{dt}}=\gamma M_{R}$$

With the translation efficiency, *γ_Rb_* ≡ *k_Rb_*/*m_R_* being a rate (unit 1/time) and *m_R_* = 7459AA, the number of amino acids per ribosome.

The increase in ribosomal mass depends in turn on how fast proteins are synthesized, and to which extent the cell is allocation translation resources toward the synthesis of novel ribosomes (the thickness of arrow 3 in Fig EV3A). We write:

$$\frac{dM_{R}}{\mathrm{dt}}=\alpha_{\mathrm{Rb}}\frac{\mathrm{dM}}{\mathrm{dt}}$$

and call *α_Rb_*, a number between 0 and 1, the allocation coefficient toward ribosome synthesis.

To understand how biomass is increasing, we next have to consider translation in more detail. Notably, the translation efficiency *γ* is not a constant rate, but it is changing with the tRNA precursor concentrations ribosomes encounter within the cells; ribosomes rely on a sufficiently high concentration of charged tRNA to work efficiently. Let us thus introduce a variable *p* describing precursor concentrations within the cell. If *p* falls to low levels, translation speeds and thus biomass accumulation drop. In a first proxy, this can be described by a simple Michaelis–Menten relation:

$$\gamma =\gamma \left(p\right)=\gamma_{\max}\frac{p}{p+p_{0}}$$

The Michaelis–Menten constant *p*_0_ can be taken from measurements characterizing how translation falls with tRNA concentrations. To calculate precursor concentrations *p*, we consider the total precursor mass *M_pc_* and compare it with the total protein mass *M* in the culture: *p* ≡ *M_pc_*/*M* (a conversion can give precursors per cell volume or dry mass). To investigate how the precursor concentrations change (over time and depending on parameters), we consider the change of total mass of precursors:

$$\frac{dM_{\mathrm{pc}}}{\mathrm{dt}}=J_{pc,\;\;in}‐\frac{\mathrm{dM}}{\mathrm{dt}}.$$

Precursor mass is given by a balance of novel precursor synthesis (flux *J_in_*) and the utilization by the ribosomes increasing the total protein mass (*dM*/*dt*). The supply of precursors depends on the joint activity of metabolic enzymes, which take up nutrients and make sure they are converted to amino acids, energy, and finally charged tRNAs (the precursors ribosomes need to grow). This is a complex process that we describe here in first order by jointly considering all metabolic enzymes as one major protein class and a simple 1^st^‐order reaction, writing *J_pc,in_* = *k_Mb_M_Mb_*. *M_Mb_* is the mass of the metabolic protein class. *k_Mb_* is an effective rate describing how fast these proteins generate precursors, which we here call *metabolic efficiency*. For the precursor mass in the culture, we thus have:

$$\frac{dM_{\mathrm{pc}}}{\mathrm{dt}}=k_{\mathrm{Mb}}\;M_{\mathrm{Mb}}‐\frac{\mathrm{dM}}{\mathrm{dt}}.$$

Similarly as for the ribosomes, the increase in metabolic proteins depends on the allocation of translation activity toward these enzymes. We write:

$$\frac{dM_{\mathrm{Mb}}}{\mathrm{dt}}=\alpha_{\mathrm{Mb}}\frac{\mathrm{dM}}{\mathrm{dt}}.$$

Here, *α_Mb_* denotes the allocation parameter toward the pool of metabolic enzymes. Notably, ribosomes can only translate one protein at a time, which leads to the overall constraint that the allocation parameters need to add up to 1. In the simplest case, assuming the cell only needs to generate metabolic enzymes and ribosomes:

$$\alpha_{\mathrm{Mb}}+\alpha_{\mathrm{Rb}}=1.$$

Since cells also need to synthesize many other enzymes needed for growth, which are not directly involved in precursor supply and translation (Scott *et al*, 2010), we extend this to:

$$\alpha_{\mathrm{Mb}}+\alpha_{\mathrm{Rb}}+\alpha_{0}=1$$

with $\alpha_{0}$ denoting the allocation toward synthesizing all other proteins. All three protein classes and the allocation of protein synthesis toward those are illustrated in Fig EV3A. The constraint is described by the relative thickness of the three arrows.

With this formulation, we can analyze the balanced exponential growth, which emerges:

$$\frac{1}{M}\frac{\mathrm{dM}}{\mathrm{dt}}=\lambda =constant.$$

When the cellular concentrations and fractions are not changing over time, $\frac{\mathrm{dp}}{\mathrm{dt}}=\frac{1}{M}\frac{dM_{\mathrm{Rb}}}{\mathrm{dt}}=\frac{1}{M}\frac{dM_{\mathrm{Mb}}}{\mathrm{dt}}=0$; and $\frac{M_{\mathrm{Rb}}}{M}=\alpha_{\mathrm{Rb}}$ and $\frac{M_{\mathrm{Mb}}}{M}=\alpha_{\mathrm{Mb}}$. The growth rate *λ* depends on the rates (translation efficiency *γ_max_* and metabolic efficiency *k_Mb_*), as well as the allocation parameters *α_Rb_* and *α_Mb_*. It can be calculated by the solution of a quadratic equation. Notably, the cell appears to control novel ribosome synthesis and adjusts the allocation parameter *α_Rb_* toward optimizing growth rates: Ribosome synthesis is regulated such that precursor levels are optimal, and ribosomes can translate close to full speed (Dai *et al*, 2016). For our purpose, this means that we know the allocation toward ribosomes for steady growth on glucose (or other carbon sources) and we can parametrize the model for steady‐state growth and subsequently extend this description to analyze growth transitions.

Modeling nutrient consumption: Up to now, we have not explicitly considered nutrient consumption but assumed that precursor influx via the metabolic enzymes is described by a constant metabolic efficiency *k_Mb_*. To consider nutrient availability, a crucial step toward describing shifts, we model the metabolic efficiency *k_Mb_* to be dependent on the nutrient concentration (say glucose, *n_glu_*) in the culture:

$$k_{\mathrm{Mb}}\left(n_{\mathrm{glu}}\right)=k_{Mb,max}\frac{n_{\mathrm{glu}}}{n_{\mathrm{glu}}+K_{M,glu}}$$

*k_Mb,max_* denotes the maximum efficiency when nutrients are not limiting. *K_M,glu_* is the Monod constant for growth on glucose. To describe the change in nutrient availability, we consider the consumption of all nutrient molecules in the culture (nutrient mass *N_glu_*) and write:

$$\frac{\mathrm{dN}}{\mathrm{dt}}={‐k}_{\mathrm{Mb}}M_{\mathrm{Mb}}/Y_{glu.}$$

Here, the yield *Y_glu_* describes the conversion from nutrients (glucose) to precursors (charged tRNA). To obtain the yield in units of amino acids, one can take measured yield value in units of dry weight per nutrient weight and then convert assuming a fraction of 60% dry‐weight content being proteins made up of amino acids with an average weight of 118.9 g/mol.

Model parameters for growth on one carbon source: To further parametrize the model, we can take translation speeds from *in vivo* measurements, and the allocation parameters from steady‐state growth analysis across growth conditions (see above). With that, the metabolic efficiency *k_Mb_* is the only parameter remaining and we adjust this parameter such that the emerging growth rate matches the one experimentally observed for growth on glucose. Parameters are provided in Appendix Table S3. The resulting dynamics is shown in Fig EV4A–E (brown dashed lines that initially follow the black lines) for a culture starting with a glucose concentration of 0.6 mM. Cells grow steadily with a constant growth rate before glucose runs out, precursor concentrations drop, and cell‐growth stops.

Modeling growth transitions: To describe growth transition from growth on glucose toward growth on acetate, we build on the formulation for growth on glucose described before and consider how cells start to express the required enzymes (e.g., *AceB*) to replenish precursors once glucose is depleted. To do this, we introduce a second class of metabolic enzymes with mass *M_Mb,ace_* (Fig EV3B). Notably, this class of enzymes includes only those enzymes, which are needed to ensure a recovery of the precursor influx (glyoxylate shunt and gluconeogenesis genes; Fig EV1B) and not those metabolic enzymes, which are also needed for growth on glucose and thus already available and working (such as TCA enzymes or enzymes of the respiratory chain to provide energy). *M_Mb,ace_* describes thus a much smaller pool of proteins than what is eventually needed for balanced growth on acetate. Since we are interested in explaining lag‐times until precursor levels recover, we here consider the requirements for those latter enzymes only indirectly by limiting the relative fraction of the enzyme class proteins (*M_Mb,ace_*/*M*) to a maximum value (more below).

With the two metabolic fluxes, the precursor turnover before and during the shifts is given by:

$$\frac{dM_{\mathrm{pc}}}{\mathrm{dt}}=k\left(n_{\mathrm{glu}}\right)\cdot M_{Mb,glu}+k_{Mb,ace}\left(n_{\mathrm{ace}}\right)\cdot M_{Mb,\;ace}‐\frac{\mathrm{dM}}{\mathrm{dt}}$$

Nutrient concentrations in the culture change accordingly:

$$\frac{dN_{\mathrm{glu}}}{\mathrm{dt}}={‐k}_{Mb,glu}\left(n_{\mathrm{glu}}\right)\frac{M_{Mb,glu}}{Y_{\mathrm{glu}}}$$

$$\frac{dN_{\mathrm{ace}}}{\mathrm{dt}}={‐k}_{Mb,ace}\left(n_{\mathrm{ace}}\right)\frac{M_{Mb,ace}}{Y_{\mathrm{ace}}}.$$

Here, the metabolic efficiencies depend on the abundance of glucose and acetate, respectively. As for growth on glucose alone, we model a Monod‐type relation with Monod constants *K_M,glu_* and *K_M,ace_* to describe how influx stops at low nutrient concentrations.

The synthesis of new metabolic enzymes depends on the allocation coefficients:

$$\frac{dM_{Mb,glu}}{\mathrm{dt}}=\alpha_{Mb,glu}\frac{\mathrm{dM}}{\mathrm{dt}}$$

$$\frac{dM_{Mb,ace}}{\mathrm{dt}}=\alpha_{Mb,ace}\frac{\mathrm{dM}}{\mathrm{dt}}.$$

Depending on the availability of nutrients, the cell is adjusting the allocation to these enzyme classes, and to model growth transitions, we thus have to formulate relations describing how the allocation coefficients depend on the availability of both nutrient sources.

The allocation to enzymes required for growth on glucose is high during steady‐state growth on glucose, but we assume that their expression reduces to lower levels when glucose concentrations drop; we thus model:

$$\alpha_{Mb,glu}={\alpha_{Mb,glu,min}+\alpha }_{Mb,glu,max}\left(\frac{n_{\mathrm{glu}}}{n_{\mathrm{glu}}+K_{M}}\right).$$

Here, $\alpha_{Mb,glu,max}+\alpha_{Mb,glu,min}$ is the same allocation coefficient we use to describe steady growth on glucose alone. In contrast, the allocation toward the required enzymes to recover precursor supply from acetate is only high once glucose runs out and this enzyme class is hardly expressed when glucose is still available. To account for this behavior, we use the following dependence on glucose concentrations:

$$\alpha_{Mb,ace}=\alpha_{Mb,ace,max}\left(1‐\frac{n_{\mathrm{glu}}}{n_{\mathrm{glu}}+K_{M}}\right)\left(1‐\frac{M_{\mathrm{ace}}/M}{\frac{M_{\mathrm{ace}}}{M}+\alpha_{Mb,ace,steady}}\right)+\alpha_{Mb,ace,preshift}\left(\frac{n_{\mathrm{glu}}}{n_{\mathrm{glu}}+K_{M}}\right).$$

That is, the synthesis of the novel metabolic enzymes required to provide precursors via acetate consumption is occurring by a certain fraction of translating ribosomes (*α_Mb,ace,max_*) once glucose is consumed. But this high rate falls again to the final steady‐state levels for growth on acetate once that fraction is reached. This limitation of the metabolic enzymes to a lower fraction allows us to indirectly consider that cells have to start synthesizing a broad class of metabolic proteins (such as TCA cycle proteins) to grow once precursor supply has been re‐established (and not only the glycolytic shunt and gluconeogenesis genes required immediately to rescue precursor influx). Finally, to study the role of pre‐shift expression, we also included an expression term $\alpha_{Mb,ace,pre‐shift}$, which describes expression when glucose is still abundant (in the reference condition, this constant is 0).

As previously, the growth kinetics is described by how the ribosomes synthesize new biomass:

$$\frac{\mathrm{dM}}{\mathrm{dt}}=\gamma \left(p\right)\;M_{R}$$

Translation and thus growth depend on precursor levels, which in turn depend on the abundance and activity of the metabolic activities.

Model parameters: We modeled growth transitions for a reference parameter set listed in Appendix Table S3. Here, we provide further context. To describe nutrient uptake toward precursor synthesis, we used yield values (*Y_glu_*, *Y_ace_*) and Monod constants ($K_{m,glu},K_{m,ace}$) known for growth on glucose and acetate. To determine the allocation parameters toward synthesis of the metabolic enzymes required to provide precursors when growing on acetate ($\alpha_{Mb,ace,preshift}$, $\alpha_{Mb,ace,max}$, $\alpha_{Mb,ace,steady}$), we used the transcriptomics measurements we collected during the shift (Fig EV5). Given the fast turnover of mRNA, these data provide a direct readout of the allocation behavior at different time points during the shift. We specifically estimated the relative mRNA fraction of all glyoxylate and gluconeogenesis genes, which are required for the continuous influx of precursors when glucose runs out (Fig 1B), and thus used 3 and 1% as reference values for $\alpha_{Mb,ace,max}$ and $\alpha_{Mb,ace,steady},$ respectively. We initially neglected pre‐shift expression levels as those are very low, $\alpha_{Mb,ace,preshift}=0$. With the allocation parameters defined, only one fitting parameter remains, the rate $k_{Mb,ace}$describing how fast metabolic enzymes recover precursors from acetate. We adjusted this rate such that the lag‐time of the modeled growth transition approximately matches the lag‐time in the experiments, ~3.5 h for the shift of WT cells (or the LacZ titration strain NQ1389 in the absence of induction) from growth in glucose to growth on acetate (Fig 1A). With these parameters, the post‐shift growth that emerges also resembles the growth rate observed during the experiments. The simulated growth transition for this reference parameter set is shown in Fig EV4A–E (black lines). Growth is fast in glucose, then stops temporarily and after a lag growth recovers by using acetate.

The delaying effect of non‐needed protein expression on growth transitions: With the formulated model and the given parameters, we can now investigate how growth transitions are changing when the cell is allocating varying fractions of its translation activity during the shift to the required metabolic enzymes. Mathematically, this means varying the allocation parameter $\alpha_{Mb,ace,max}$. The results are shown in Fig EV5F–J. The lag‐time falls strongly with a higher allocation toward the required enzymes, with the reciprocal relation shown in Fig 3B. As discussed in Fig EV5, different allocations lead to varying drops of precursor levels during the shift, which changes the ability to recover growth. To probe this prediction of the model, we compared the predicted change in lag‐times with those experimentally observed when overexpressing the non‐needed gene lacZ (experimental data shown in Fig 2). To compare data and model predictions, we plotted the lag‐times versus the fold change of required genes, which we calculated from the allocation parameter $\alpha_{Mb,ace,max}$ and the *aceB* qPCR measurements for the model and experiments, respectively; see Fig 3C. The observed trend is captured by the model without further fitting.

In Appendix Supplementary Text, we further analyze how the duration of growth arrest (lag‐time) falls with the pre‐expression of genes required to maintain a precursor flux after the shift (parameter $\alpha_{Mb,ace,pre‐shift})$.

## Author contributions

RB and JC conceptualized and designed the experiments. RB, JC, and RTD performed the experiments. JC performed mathematical modeling. RB, JC, and TH analyzed the data. RB and JC wrote the manuscript. JC and TH acquired funding.

## Conflict of interest

The authors declare that they have no conflict of interest.

## Supporting information



Appendix

Expanded View Figures PDF

Source Data for Expanded View

Source Data for Figure 1

Source Data for Figure 2

Source Data for Figure 4

Source Data for Figure 5

## Data Availability

The RNA‐Seq data generated in this study are available via Gene Expression Omnibus (GEO), accession number GSE185426 (https://www.ncbi.nlm.nih.gov/geo/query/acc.cgi?acc=GSE185426). The computational code of the model is available via GitHub at https://github.com/jonascremer/lagtimemodeling.
