# Supplementary material for: Suboptimal resource allocation in changing environments constrains response and growth in bacteria
Source: Mol Syst Biol. 2021 Dec 20;17(12):e10597. doi: 10.15252/msb.202110597 (PMC8687047; doi:10.15252/msb.202110597)
Supplement: Supplementary file 1 — Appendix [file MSB-17-e10597-s002.pdf]

Suboptimal resource allocation in changing environments constrains response and growth in bacteria

Rohan Balakrishnan, Roshali T. de Silva, Terence Hwa, Jonas Cremer

Table of Contents

APPENDIX TEXT 1 2

ADDITIONAL MODELING CONSIDERATIONS 2
1.1. VARIATION OF THE PRE-SHIFT EXPRESSION OF REQUIRED PROTEINS 2
1.2 RIBOSOME CONTENT 3

APPENDIX TABLES 3

APPENDIX TABLE S1: STRAINS USED IN THIS STUDY 3
APPENDIX TABLE S2: PRIMERS USED IN THIS STUDY 4
APPENDIX TABLE S3: MODEL PARAMETERS 4

REFERENCES 6

Appendix Text 1

Additional modeling considerations

We provide here additional considerations of the model we introduced to describe growth transitions and the impact of required and non-required gene expression on duration of growth arrest (lag-times). In Section 1.1. we discuss how lag-times are changing with the pre-expression of required proteins already before the shift. In Section 1.2. we comment why we did not explicitly model the expression of ribosomal proteins (and other proteins required for protein synthesis) during the shift.

1.1. Variation of the pre-shift expression of required proteins

Our transcription results show that the enzymes required to recover growth once glucose runs out are hardly expressed when glucose is still abundant (**Fig. EV6**), suggesting that pre-shift expression levels do not play a major role in shaping lag-times for WT *E. coli* cells for these growth-conditions. But a higher pre-shift expression might occur in specific growth conditions and has been shown to support faster transition in a synthetic strain were pre-expression of the required genes *aceB/aceA* was controlled by an inducer construct(Basan *et al*, 2020). To investigate the role of pre-shift expression compared to expression during the shift we determined the emerging lag-times when both, the allocation before the shift ($\alpha_{Mb,ace,preshift}$) and during the shift ($\alpha_{Mb,ace,max}$) are varied (Appendix Figure 1 below).

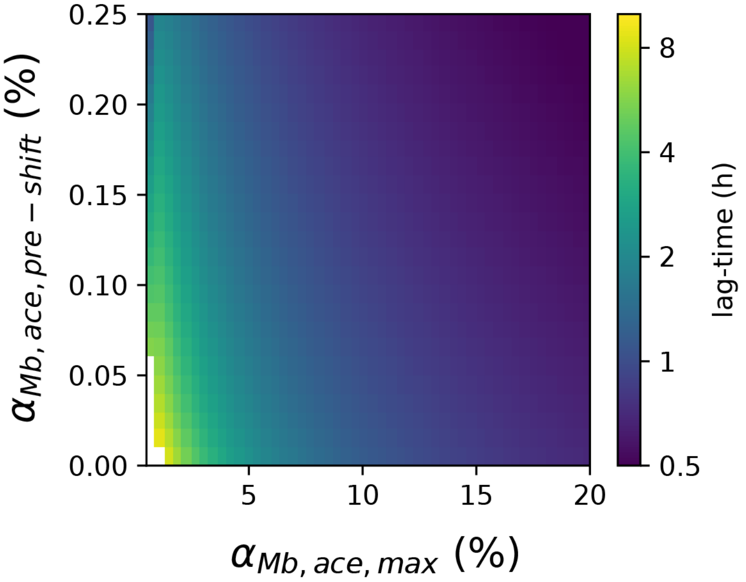

Appendix Figure 1: Lag-times when varying pre-shift and during shift expression of the genes required to recover precursor influx when growing on acetate. We varied both allocation parameters $\alpha_{Mb,ace,preshift}$ and $\alpha_{Mb,ace,max}$ independently. Pre-shift

expression of the enzymes can clearly lead to a faster lag-times. Notably, however, lag-times can also be short without pre-shift expression if cells response to the shift with a strong expression of required genes instead of the expression of other genes. The previously described tradeoff between fast pre-shift growth and fast shift thus appears to be a secondary tradeoff effect important when there are long lag-times in the first place because cells response by the expression of non-required enzymes.

1.2 Ribosome content

Up to now, we have not explicitly modeled the change of ribosomes synthesis during the shift. An explicit consideration of ribosome synthesis is not required to investigate the origin of lag-times since ribosomes are highly abundant at the moment glucose runs out and they do not contribute to a bottleneck in precursor influx. And since ribosome synthesis abruptly stops at the shift (**Fig. EV5G**) we do not have to consider the role of novel ribosome synthesis as a process competing for the same limited precursors required to synthesize the required enzymes to recover metabolic flux. Indeed, when explicitly modeling the change of novel ribosome synthesis lag-times do not change (data not shown). Particularly, we modeled that ribosomes are only synthesized when precursor levels are above a threshold value, in line with what is currently known about the control of ribosome synthesis mediated by central regulators like the alarmone ppGpp. The allocation we modeled is:

$$\alpha_{Rb}(p) = \alpha_{Rb,steady} \cdot p/(p + p_0).$$

APPENDIX TABLES

Appendix Table S1: Strains used in this study

Strain name	Description	Details (genotype, plasmid)	Ref
NCM3722	Wild type (K12), genetic background for all strains used in this study		(Soupene *et al*, 2003)
NQ1389	*lacZ* over-expression strain	Ptet-tetR on pZA31; Ptetstab-lacZ on pZE1	(Scott *et al*, 2010)
NQ1350	*aceBA* induction	aceBA promotor controlled by Ptet	(Basan *et al*, 2020)
GE029	*ΔfliC*		This study

NQ1225	*ΔflhD*		(Basan *et al*, 2015)

Appendix Table S2: Primers used in this study

Name	Sequence	Usage
aceA-qpcr-F	GCGTTGGGAAGGCATTACTCGC	qPCR
aceA-qpcr-R	GCCTGACCGCCAGTCAGTGC	qPCR
aceB-qpcr-F	GGCAACAACAACCGATGAACTGGC	qPCR
aceB-qpcr-R	GCTCAGTCAGAAATTCTACCGC	qPCR
lacZ-qpcr-F	GGCAATTTAACCGCCAGTCAG	qPCR
lacZ-qpcr-R	GTGCACGGGTGAACTGATC	qPCR
16S-qpcr-F	CCTAGGCGACGATCCCTAGC	qPCR
16S-qpcr-R	CATACACGCGGCATGGCTGC	qPCR
fliC-F	CAGGGTTGACGGCGATTGAG	Confirmation KO
fliC-R	CAATTTGGCGTTGCCGTCAG	Confirmation KO
k1	CAGTCATAGCCGAATAGCCT	Confirmation KO
k2	CGGTGCCCTGAATGAACTGC	Confirmation KO
kt	CGGCCACAGTCGATGAATCC	Confirmation KO

Appendix Table S3: Model parameters

Symbol	Description	Value
$\alpha_{Rb,glu}$	Allocation to translation (ribosomes)	0.2
$\alpha_{Mb,glu}$	Allocation to metabolic proteins	0.45
$k_{Mb,glu}$	Metabolic efficiency glucose	$2.4\ 1/h$
Y_{glu}	Yield in glucose	$0.377\ OD_{600}/mmol$

$K_{M,glu}$	Monod constant, glucose	$5\mu mol$
$\alpha_{Rb,ace,steady}$	Allocation to translation (ribosomes)	0.2
$\alpha_{Mb,ace,max}$	Allocation to required enzymes at shift (or varied)	0.03
$\alpha_{Mb,ace,steady}$	Allocation to required enzymes at shift at steady growth	0.01
$k_{Mb,ace}$	precursor-rate for bottleneck enzymes (when other metabolic enzymes are ready)	$30\ 1/h$
Y_{ace}	Yield in acetate	$Y_{glu}/2$
$K_{M,ace}$	Monod constant	$5\mu mol$
	Conversion between OD and AA. Based on known conversion between OD and biomass, 0.63% of dry mass being proteins. More details provided at GitHub.	$7.69\ 10^{-18}\ OD_{600} \cdot ml/AA$
f_{Rb}	fraction active ribosomes	0.65
k_{Rb}	Max. translation elongation rate	$20\ 1/s$
p_0	Michaelis Menten constant for translation. In units of charged tRNA per mass amino acids.	0.026

REFERENCES

Basan M, Honda T, Christodoulou D, Hörl M, Chang Y-F, Leoncini E, Mukherjee A, Okano H, Taylor BR, Silverman JM, *et al* (2020) A universal trade-off between growth and lag in fluctuating environments. *Nature*: 1–5

Basan M, Zhu M, Dai X, Warren M, Sévin D, Wang Y & Hwa T (2015) Inflating bacterial cells by increased protein synthesis. *Mol Syst Biol* 11: 836–836

Scott M, Gunderson CW, Mateescu EM, Zhang Z & Hwa T (2010) Interdependence of Cell Growth and Gene Expression: Origins and Consequences. *Science* 330: 1099–1102

Soupene E, Heeswijk WC van, Plumbridge J, Stewart V, Bertenthal D, Lee H, Prasad G, Paliy O, Charernnoppakul P & Kustu S (2003) Physiological Studies of Escherichia coli Strain MG1655: Growth Defects and Apparent Cross-Regulation of Gene Expression. *J Bacteriol* 185: 5611 5626
