# Supplementary material for: Suboptimal resource allocation in changing environments constrains response and growth in bacteria
Source: Mol Syst Biol. 2021 Dec 20;17(12):e10597. doi: 10.15252/msb.202110597 (PMC8687047; doi:10.15252/msb.202110597)
Supplement: Supplementary file 2 — Expanded View Figures PDF [file MSB-17-e10597-s007.pdf]

Expanded View Figures

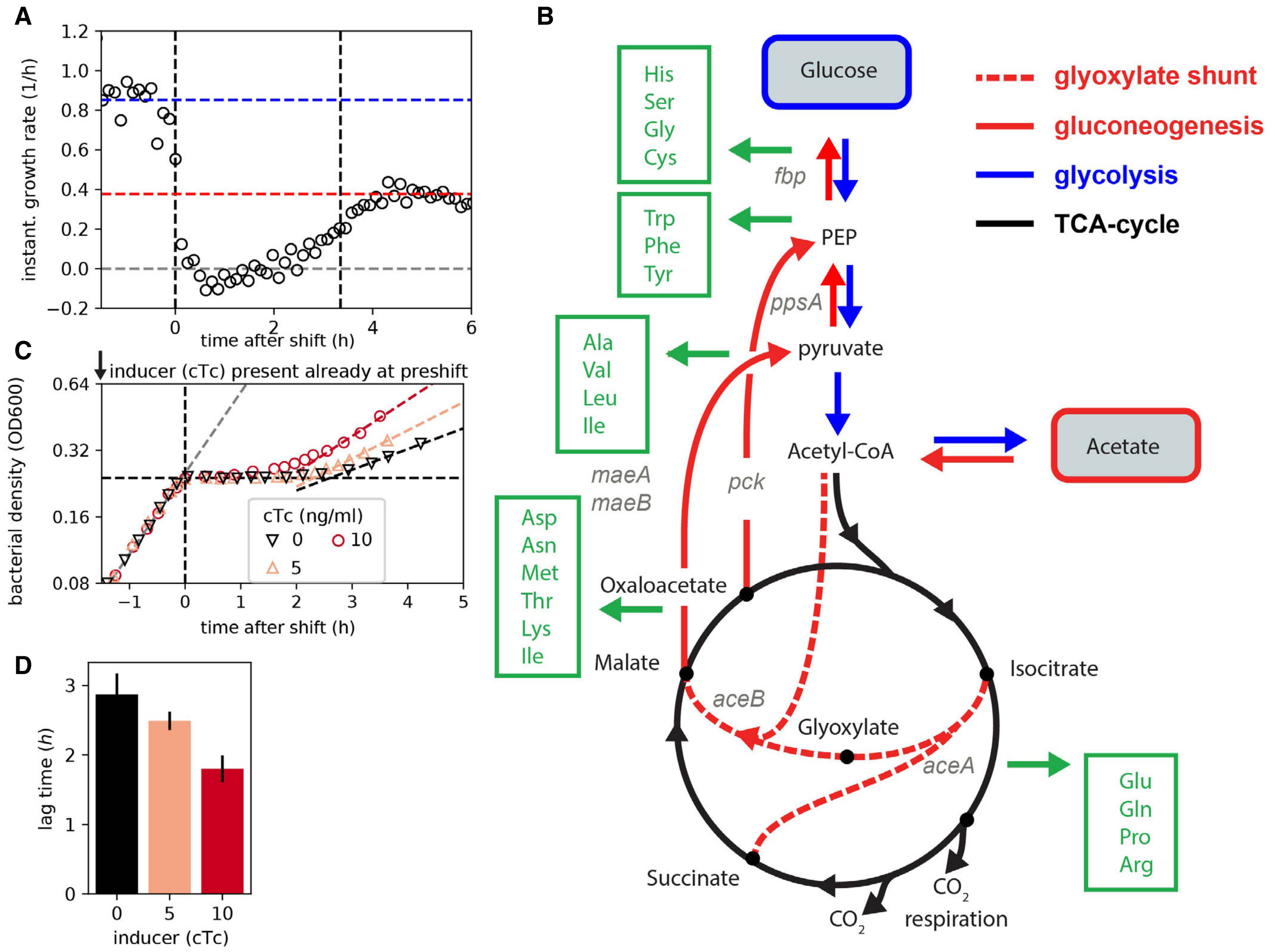

Figure EV1. Diauxic growth on glucose and acetate: transition kinetics and metabolic requirements.

A Instantaneous growth rate of the WT (NCM3722) is derived by calculating the derivative of the growth rate divided by the optical density (growth curve shown in Fig 1A). Cells first consume glucose and grow exponentially at a rate of ~0.9/h (blue horizontal line). Following glucose depletion (defined as time 0; left vertical dashed line), the instantaneous growth rate immediately falls and growth stops. After a phase of no growth, growth gradually begins to approach a rate of 0.4/h, the steady-state growth rate for growth on acetate (red horizontal line). Right vertical dashed line indicates the time of growth recovery as determined by the fitting of an exponential curve (see Lag-time quantification in Materials and Methods).

B To resume growth on acetate following glucose depletion, the supply of amino acids, the major precursors required for biomass synthesis, must be re-established. The various nodes along the central carbon metabolism that branch into the synthesis of different amino acids are indicated in green. For the successful recycling of the 2 carbon molecules per acetate into amino acid precursors, the most essential step is the activation of the glyoxylate shunt (Wolfe, 2005): To prevent the loss of the 2 CO_2 molecules occurring along the TCA cycle, the carbon flux has to be bypassed. Instead of being converted to alpha-KG, isocitrate is split into succinate and glyoxylate by the enzyme isocitrate lyase (*AceA*). Glyoxylate is then converted to malate by the malate synthase (*AceB*). Succinate and malate generated as a result of the shunt subsequently fuel gluconeogenesis (*MaeA*, *MaeB*, *Pck*, *PpsA*), making available the carbon precursors required for the synthesis of many amino acids (green arrows). Besides amino acids as precursors, protein synthesis also requires substantial amounts of energy, primarily to charge tRNA and drive translation. However, energy supply is unlikely to be the major bottleneck during this shift since cells already express and utilize TCA enzymes during the pre-shift growth, which can thus ensure a continuous production of ATP over the course of the transition.

C, D Effect of maintaining pre-expressed aceBA reserves on growth transitions. Diauxic transitions of NQ1350 in which the native aceBA promoter is replaced by the inducible P_{tet} promoter are shown when 0, 5, or 10 ng/ml inducer cTc is added to the medium already at the pre-culture stage, well before glucose depletion. Bar plot (D) shows the mean lag-times of $N = 2$ biological replicates. Error bars indicate SD.

Source data are available online for this figure.

 © 2021 The Authors

 © 2021 The Authors

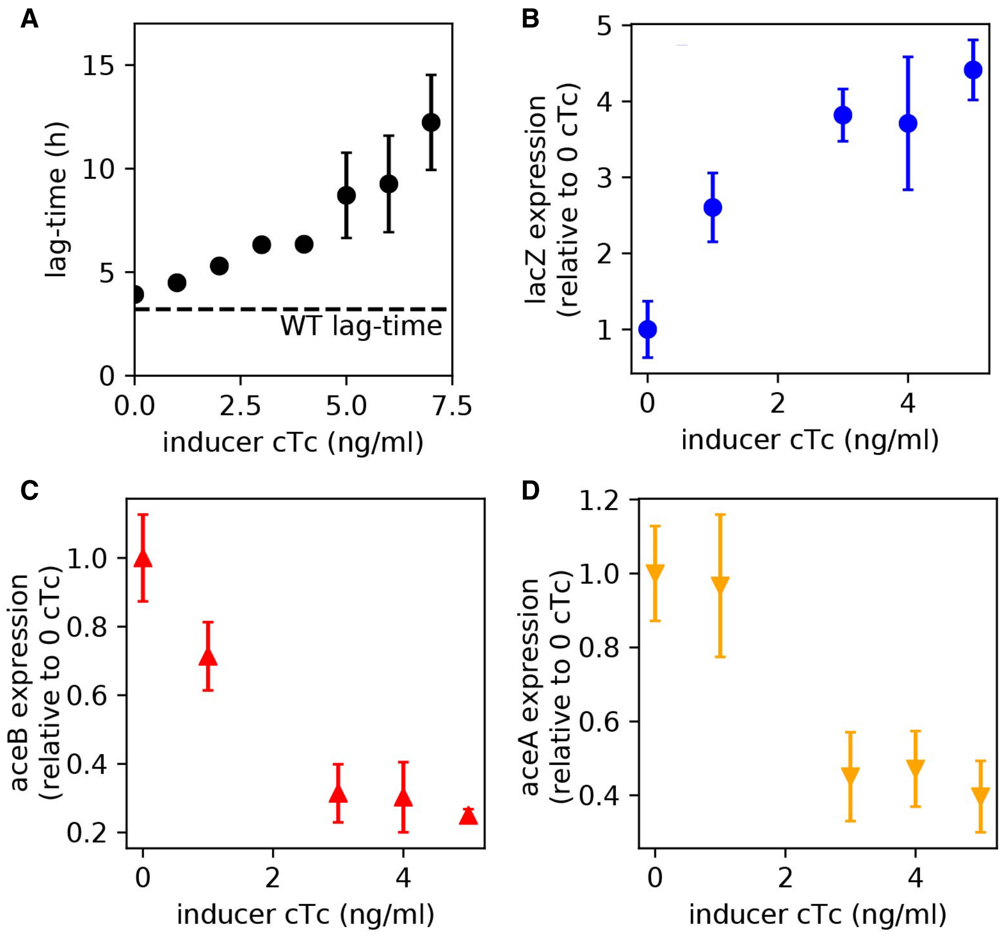

Figure EV2. Lag-times and gene expression when overexpressing lacZ.

A Increase in lag-times with increasing inducer levels (strain NQ1389). The inducer chlortetracycline (cTc) is added when glucose runs out (Fig 2A, time = 0). Dashed horizontal line indicates lag-time for the WT strain (NCM3722).

B–D mRNA levels of *lacZ*, *aceB*, and *aceA* at different cTc levels are quantified by qPCR 10 min after the depletion of glucose. mRNA levels of each gene are normalized to the 16S rRNA level, which is known to remain constant during the lag phase (Bosdriesz *et al*, 2015; Erickson *et al*, 2017). These normalized expression levels are shown relative to the expression level in the absence of induction (0 cTc).

Data information: Mean of $N = 3$–5 biological repeats shown. Error bars indicate SD.
Source data are available online for this figure.

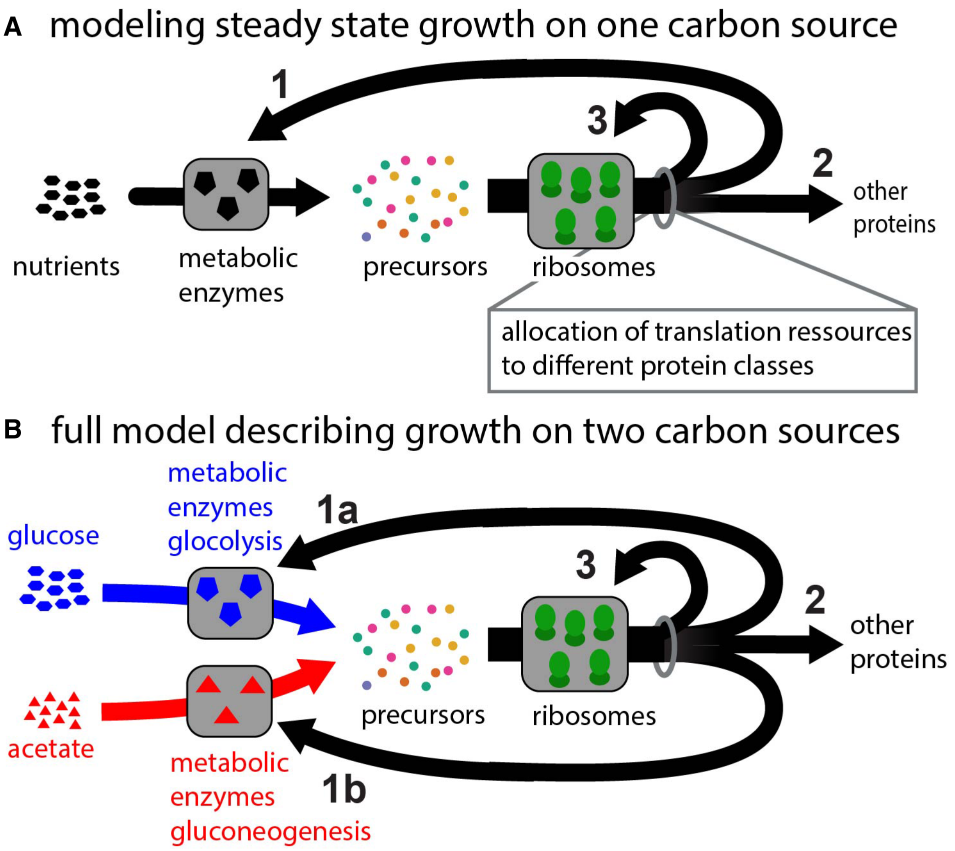

Figure EV3. Modeling growth in changing environments.

A, B To model growth during transitions, we here build on replicator/allocation models, which have been established previously for growth in steady-state conditions and for specific shifts. Replicator/allocation models consider protein synthesis by ribosomes toward different proteins (Scott *et al*, 2014), and central to their approach is the allocation of ribosome activity toward the synthesis of different proteins. To illustrate the concept, we here consider steady growth on one carbon source first (A). The pool of ribosomes is allocated toward different protein classes such as the proteins for novel ribosomes (A, arrow 3), metabolic proteins needed to provide the precursors when utilizing the carbon source (A, arrow 1) and other proteins (A, arrow 2). Growth depends on the availability of nutrients and how the ribosomal activity is allocated to the different protein classes: High growth rates are achieved with allocation ratios that balance precursor influx provided by the metabolic enzymes and their utilization by ribosomes, such that as many ribosomes as possible can translate at maximum speed. This logic is formulated mathematically in Materials and Methods, and Appendix Supplementary Text. Notably, for *Escherichia coli* the allocation of ribosomal activity toward the synthesis of new ribosomes follows indeed a close to optimal regulation scheme preventing the synthesis of idle ribosomes (as manifested in the ribosome content changing with growth rate). To describe growth during shifts, we extend this modeling approach and explicitly consider glucose and acetate as two nutrient sources (B). In this case, two metabolic protein classes (arrows 1a and 1b), which provide the precursors when cells grow on the two carbon sources (blue and red arrows) and perform glycolysis (blue) or gluconeogenesis (red), respectively. As such, this model structure shares similarities with recent modeling approaches to describe growth during nutrient shifts (Molenaar *et al*, 2009; Erickson *et al*, 2017; Korem Kohanim *et al*, 2018). However, our approach is distinguished from those studies by the inclusion of two key aspects, which are central to the cellular response during growth shifts: (i) We consider the highly responsive regulation of transcription and integrate our transcription measurements (Fig EV5), which quantifies the immediate expression response of the cell during the shift. (ii) We explicitly vary the allocation toward other proteins shift (arrow 2) during the shift, thereby bringing the focus of the study to the allocational constraints acting during the response itself. Notably, right after the shift the model simplifies to the scenario shown in Fig 3A and the consideration of metabolic enzymes (arrows 1 and 2) together with the precursors required to drive novel protein synthesis is sufficient to investigate how long lag-times can emerge and how they relate to the expression of non-required proteins. The mathematical formulation of the full model and additional context is provided in Materials and Methods, and results for the switch from growth on glucose to growth on acetate are provided in Fig EV4. The central model output, the curve describing how lag-times decrease with an increasing allocation to required proteins, is shown in Fig 3B.

 © 2021 The Authors

Figure EV4. Modeling growth transitions and the consequence of non-required protein expression.

Based on our model of growth transitions (Fig EV3, Materials and Methods), we analyzed growth kinetics during the transition from growth on glucose to growth on acetate.

A–E Change of major model variables (cell density, nutrients, precursors, and required metabolic enzymes) during the shift for a reference condition with a specific allocation toward the metabolic enzymes required for growth on acetate. Growth and nutrient abundance: When glucose is available, cells grow fast (A, blue region) consuming only glucose, and not acetate (B, blue region). Following glucose depletion, precursors fall dramatically (D, gray region) and growth thus stops (A and C, gray region). Cells then only slowly synthesize the required metabolic enzymes such as AceB (E), which then slowly support higher precursor concentrations (D, gray region). The case where no metabolic proteins for growth on acetate are synthesized is shown for comparison (D, dashed line); precursor concentrations continue to fall. Growth only recovers to post-shift growth rates (A and C, red regions) once precursors reach concentrations comparable again to the Michaelis–Menten constant (D, dashed horizontal line), which describes the concentration of precursors above which ribosomes can work efficiently with close to maximum translation rates. The growth recovery is slow and spans several hours since cells are trapped in a state where precursor concentrations and the abundance of the metabolic enzymes that can generate new precursors are both low. Accordingly, novel metabolic proteins can only be generated slowly and precursor concentrations thus also recover slowly.

F–J Effect on changing allocation toward the metabolic enzymes required for growth on acetate. Plots show the same variables as in (A–E) but for different allocational behavior toward the synthesis of novel proteins required during the shift (model parameter $\alpha_{\mathrm{Mb,ace,max}}$ describing the allocation of overall transcription (mRNA fraction) to the required enzymes; see color legend). A higher allocation toward the metabolic enzymes (darker colors) substantially decreases the lag during the growth transition as it leads to much faster accumulation of required metabolic enzymes (J), preventing a dramatic decrease in precursor concentrations right after the shift (I). This thus also leads to a much faster recovery of growth (I). Growth stops once acetate is also consumed (G, H). The change in lag-times for different allocations toward the required metabolic enzymes is shown in Fig 3B.

Data information: All times shown are relative to the time point where glucose is depleted, and colored regions in (A–E) indicate different growth phases as defined by the intersection of exponential growth curves (dashed lines in A and F) and the density values during the shift, following what was done for the experiments (Figs 1A and EV1A). Model parameters as listed in Appendix Table S3.

© 2021 The Authors

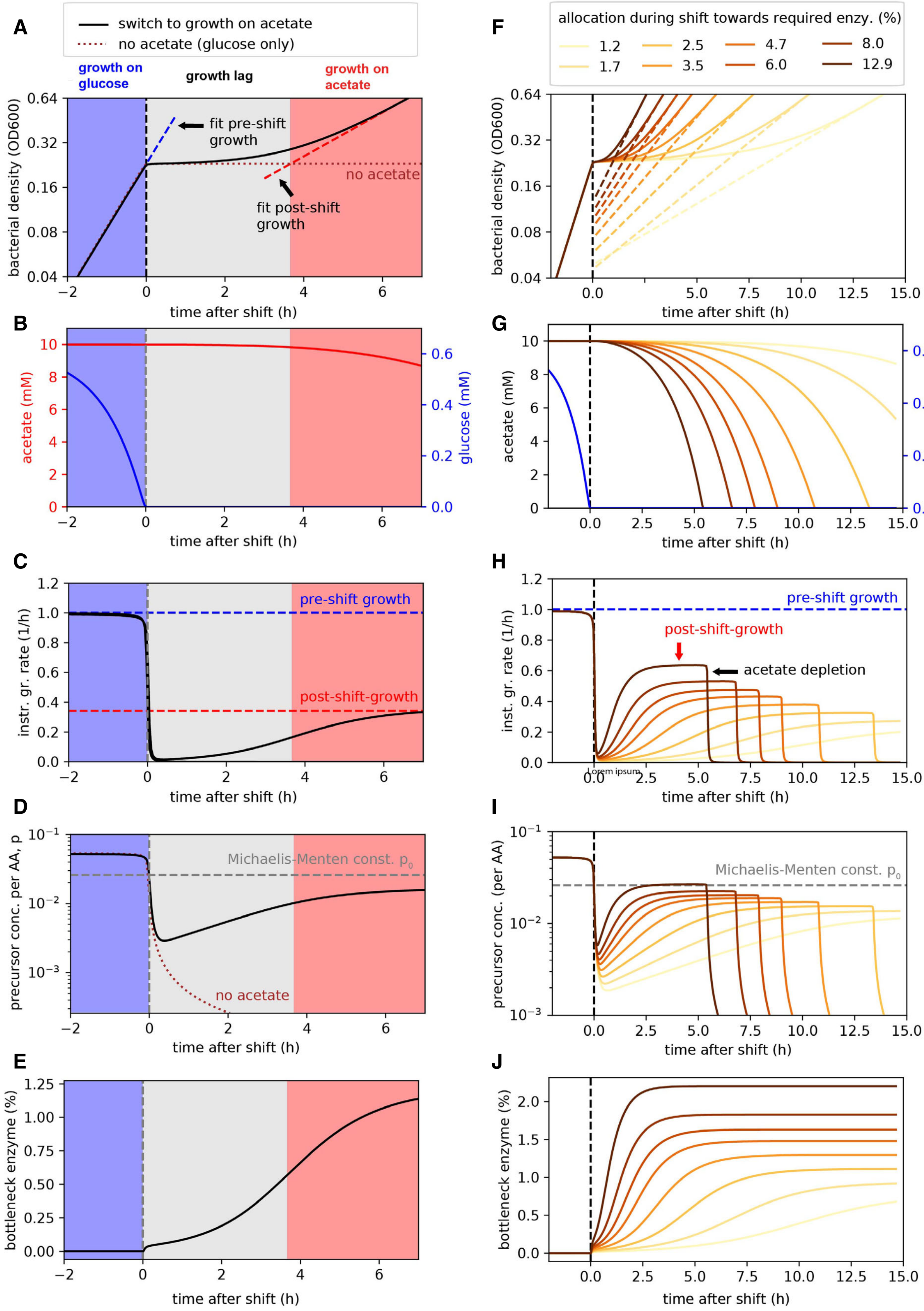

Figure EV4.

 © 2021 The Authors

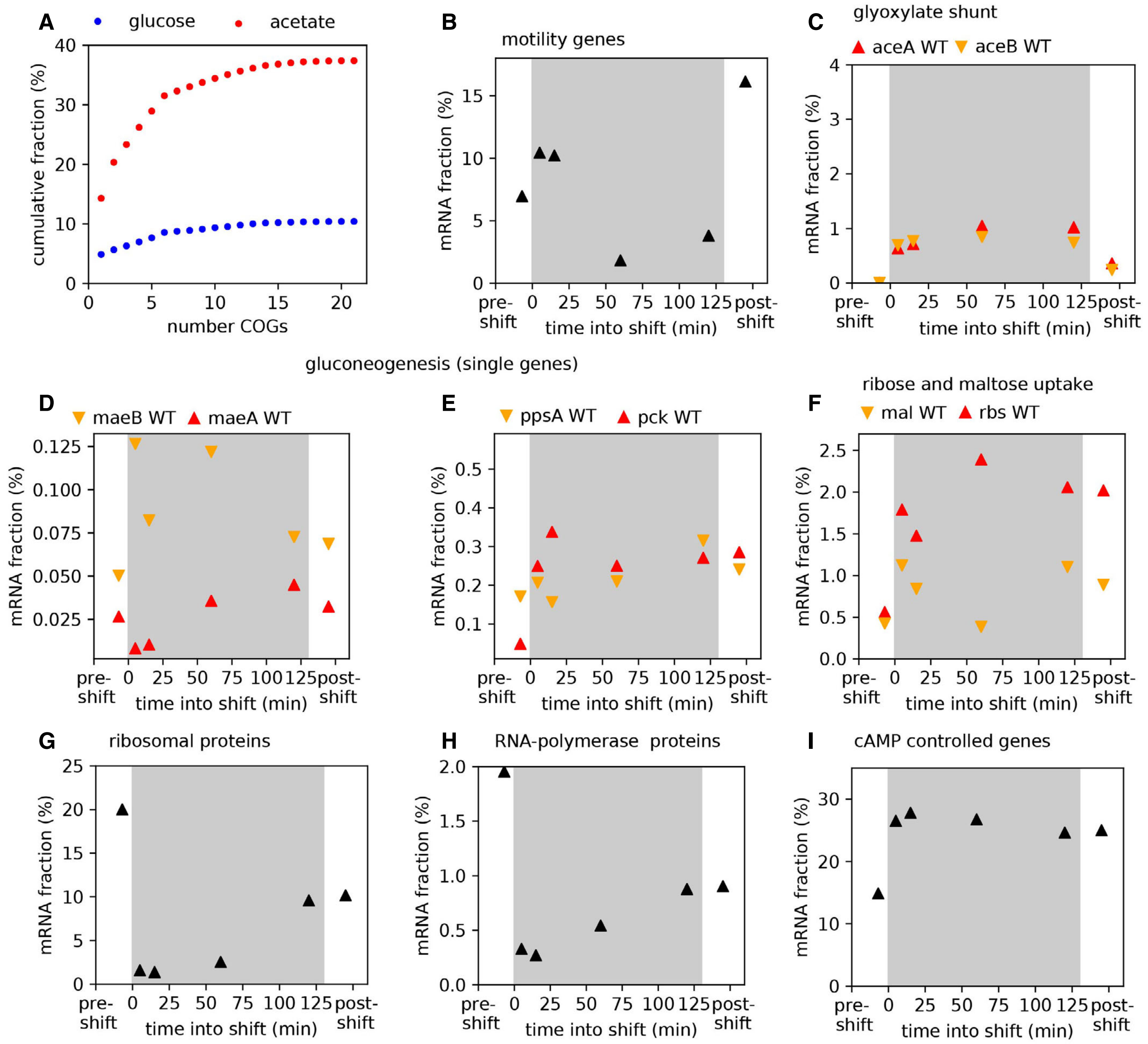

Figure EV5. Gene expression during the transition.

A Fractions of genes upregulated in acetate (red) compared with glucose (blue), shown as cumulative sum over most expressed functional groups. Data are derived from transcriptomics measurements (RNA-Seq) using adjusted clusters of orthologous groups (COGs). The five most expressed functional groups, shown in Fig 4B during the shift, cover most of the genes that are upregulated.

B–I mRNA abundance for different genes and gene groups during the diauxic shift from glucose (pre-shift) to acetate (post-shift), using the RNA-Seq measurements. (B) Motility genes (defined in (10), consisting of *fliC* encoding the major structural component of the flagella and different motor genes) are heavily expressed during the shift. (C) Glyoxylate shunt genes (detailed version of Fig 5D) are upregulated. (D, E) The upregulation of different gluconeogenesis genes. (F) *rbs* and *mal* genes (detailed version of Fig 5C) are upregulated during the shift. (G, H) Expression of ribosomal protein (summed over all *rpl*, *rpf*, and *rpm* genes) and RNA polymerase genes (*rpoA,B,C*) during the shift. Within 5 min, these genes are repressed, confirming a responsive downregulation mechanism. (I) The expression of genes known to be under the control of the second messenger cAMP (Kalisky *et ai*, 2007). This group of genes is upregulated during the shift. All data are derived from the same RNA-Seq dataset taking the relative fraction of different genes (and their sum when groups of genes are considered). Series of measurements during the shift was performed once and with the WT strain NCM3722.

Source data are available online for this figure.

© 2021 The Authors

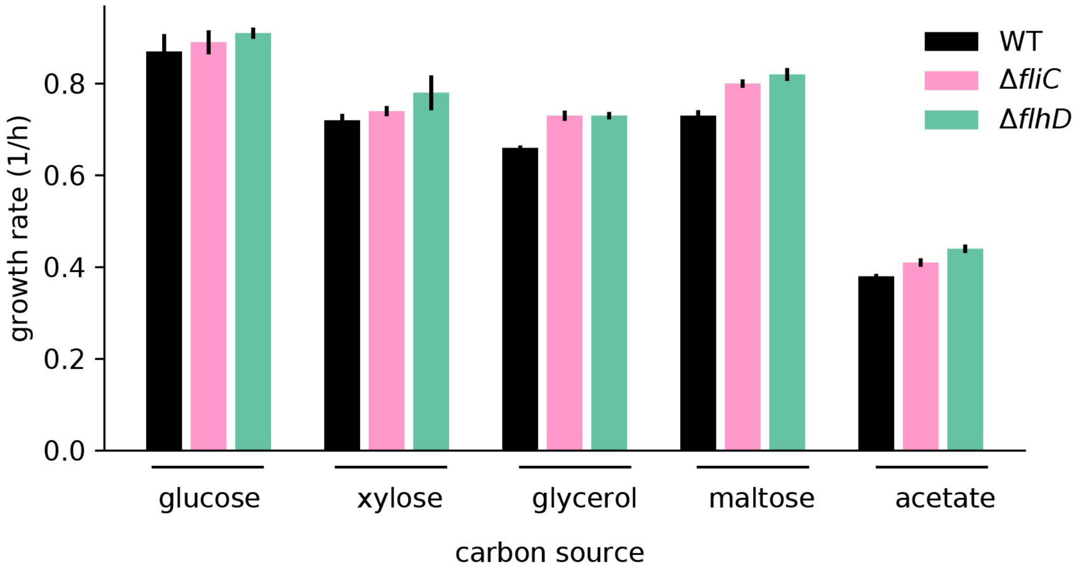

Figure EV6. Balanced growth of deletion strains.

Steady growth of WT and motility deletion strains *ΔfliC* and *ΔflhD* (strains listed in Appendix Table S2). Growth with 20 mM glucose, 20 mM xylose, 20 mM glycerol, 20 mM maltose, and 30 mM acetate. Data for growth on glucose and acetate as shown in Fig 4C. Mean of $N = 4$ biological repeats are shown. Error bars indicate SD. Differences in steady-state growth rate between WT and the mutants are significant in each case besides glucose (P-value < 0.05, except for ΔfliC in xylose which has a P-value of 0.07). All indicated P-values were computed by a two-sample t-test.

Source data are available online for this figure.

 © 2021 The Authors
