# Supplementary material for: Suboptimal resource allocation in changing environments constrains response and growth in bacteria
Source: Mol Syst Biol. 2021 Dec 20;17(12):e10597. doi: 10.15252/msb.202110597 (PMC8687047; doi:10.15252/msb.202110597)
Supplement: Supplementary file 8 [file MSB-17-e10597-s008.pdf]

molecular systems biology

Suboptimal resource allocation in changing environments constrains response and growth in bacteria

Rohan Balakrishnan, Roshali de Silva, Terence Hwa, and Jonas Cremer
DOI: 10.15252/msb.202110597

Corresponding authors: Jonas Cremer (jbcremer@stanford.edu) , Rohan Balakrishnan (rbalakrishnan@ucsd.edu)

Review Timeline:

Submission Date:	25th Jul 21
Editorial Decision:	17th Sep 21
Revision Received:	4th Nov 21
Editorial Decision:	29th Nov 21
Revision Received:	29th Nov 21
Accepted:	1st Dec 21

Editor: Maria Polychronidou

Transaction Report:

(Note: With the exception of the correction of typographical or spelling errors that could be a source of ambiguity, letters and reports are not edited. Depending on transfer agreements, referee reports obtained elsewhere may or may not be included in this compilation. Referee reports are anonymous unless the Referee chooses to sign their reports.)

RE: MSB-2021-10597, Suboptimal resource allocation in changing environments constrains response and growth in bacteria

Thank you again for submitting your work to Molecular Systems Biology. We have now heard back from two of the three referees who agreed to evaluate your study. Unfortunately, after several reminders we have not received a report from reviewer #2. In the interest of time and given that the recommendations of the other two reviewers are similar, we have decided to proceed with making a decision based on the two available reports. As you will see below, the reviewers acknowledge that the study is a relevant contribution to the field. They raise however a series of concerns, which we would ask you to address in a revision.

I think that the reviewers' recommendations are clear and therefore it is not required to repeat the points listed below. All issues raised by the reviewers need to be satisfactorily addressed. In line with the comments of reviewer #3, we would ask you to make sure that the model and data have been made publicly available and described in the "Data Availability" section (see further details below). Please contact me in case you would like to discuss in further detail any of the issues raised.

Reviewer # 1:

Balakrishnan et al. presents an interesting study investigating the cause of a lag time during diauxic shift of E. coli. During such a growth transition, cells start to produce enzymes to utilize acetate, and the question is whether cells produce enough of these enzymes to facilitate rapid transition and thereby reduce the lag time. The authors showed that decreasing the expression of a key operon aceBA increases the lag time, and that the same can be achieved by overexpression of an unnecessary gene. These results suggest that there is an inherent competition between the expression of different genes, which the authors modeled theoretically showing that suboptimal allocation of the transcriptome can lead to a prolonged lag time. As some of the most induced genes during the growth transition are flagellum genes that seem unnecessary for the specific environment, the authors created strains lacking such responses and show that the lag time can be reduced from the wildtype.

This is an interesting work showing that the transcriptome response during growth transition may not be optimized for fitness. The data are well presented and support their main model. I have several comments that the authors may consider addressing.

1. Does overexpression of aceBA ever reduce the lag time to be shorter than that of the wildtype? The authors used a strong induction system to show that the lag time is sensitive to the level of aceBA. But even at the highest induction level shown, the lag time appears to be still longer than the wildtype one. Is the maximum induction level higher than the level of aceBA in the wildtype? If so, it is interesting, and potentially puzzling, that the increased transcriptional activity of this gene cannot outcompete the resources spent on the other genes to reduce the lag time.

2. The theoretical model makes quantitative predictions on how much lag time should be observed at a given allocation to the required enzymes (Fig 3B). I was surprised that there was no comparison between experimental data and this prediction, even though the data already exist in Figure 4. The model feels unnecessary if the quantitative predictions are not tested.

3. The 'wildtype' E. coli exists in many different motility genotypes, with many different mutations that stimulate flagellum genes to different degrees (https://journals.asm.org/doi/10.1128/JB.00455-15). It would be interesting to explore whether the strain used in this study has the highest level of unnecessary motility gene expression during diauxic shift. If most wildtype isolates behave similarly, the results could strengthen the claim that the response is not optimized for the growth transition.

4. In the abstract, the sentence that "our findings highlight that cells do not optimize growth in the given environment ..." seems somewhat misleading. The focus of this study is at changing environments and not a given environment. The former is history-dependent whereas the latter implies a steady-state that is history-free. I suggest changing "in the given environment" to "under changing environments."

Reviewer #3:

Summary

The study by Balakrishnan and co-authors sheds light on the question why E. coli requires a several hour long lag-phase to make the transition from a high nutrient quality substrate, glucose, to a less-preferred substrate, acetate. Using knock-out and overexpression strains, transcriptional profiling, and resource allocation modeling, the authors show that this transition is not growth-optimal due to a diversified gene expression program. This program prevents the timely production of required enzymes and leads to a long lag phase. The lag phase can be tweaked by adding or removing the expression of genes that result in "idle" proteins.

General remarks

This study advances our knowledge of growth shifts by highlighting the importance of timely production of the required enzymes, and how other cell functions drain resources from production of these enzymes. The study adds more evidence for the hypothesis that bacteria are evolutionarily optimized for metabolic flexibility, e.g. by prioritizing nutrient scavenging (motility) over quick transition towards a specific carbon source. The study is highly interesting, well-written and experiments are sound and support the drawn conclusions. However, there are also several weaknesses that should be addressed: The authors might consider to add one particular experiment in order to prove that acetate metabolism transcripts are really the limiting factor for lag phase duration (see major comments). There are problems with some figures where the conclusions do not match the presented data (most likely because of trivial errors). Generally, the level of detail for reporting data and statistical methods is below standard and can certainly be improved by addressing some of the comments outlined below.

Major points

P3, l68: The authors write that "Lag times are greatly reduced when increasing amounts of the inducer chlorotetracycline (cTc) are added at the moment of glucose depletion (Figure 1D)."
This statement is somewhat misleading, as the lag time at full induction (100 ng/mL cTc) seems to be identical with the lag time when using the natural ace promoter, about 3.5 h. Judging from figure 1D, it is not obvious that lag time can be reduced, but it can certainly be prolonged. The authors need to clarify what the reference is when stating that lag time can be reduced, particularly because this is used to motivate the subsequent study of resource allocation during the lag phase.

In Figure 2 and in text page 3, line 82+, the authors nicely show that lag phase increases proportionally with lacZ expression, and aceA/B down-regulation. The authors attribute the lag-phase mechanistically to the reduced transcript concentration of acetate-metabolizing enzymes. However, is it not possible that the increased lag phase is a product of several overlaying effects all originating from lacZ-expression? With the assumption that protein pool size is constant but limited, we can think of:
- more lacZ transcripts, less ace transcripts (explicitly mentioned)
- lacZ dummy protein reducing ribosomal proteins needed to translate acetate metabolism enzymes (not explicitly mentioned)
- lacZ dummy protein reducing other metabolic enzymes apart from acetate metabolism (not explicitly mentioned)
Maybe the authors can clarify if and how these indirect effects of lacZ expression could explain the observed results, or how they can be untangled.

The authors show that deletion of "idle" proteins (motility functions) leads to increased growth rate and decreased lag phase during acetate transition. Page 5, line 158 reads: "The increase of growth was also observed for other carbon sources besides acetate (Fig. S6) and provides direct support for the idea that gene regulation is not optimized for steady state growth."
I just want to point out that non-optimality of gene expression for steady-state growth was also shown in other organisms by now, apart from E. coli. For example, streamlining a Pseudomonas putida strain by deleting motility genes increased growth rate and stress tolerance (Martinez-Garcia et al., Env Microb, 2013). In photosynthetic cyanobacteria, the light harvesting machinery was identified as a major protein burden that is idle in high light conditions, leading to non-optimal steady state growth in this condition (Jahn et al., Cell Reports, 2018. Full disclosure: I am an author of this paper). The authors may consider including these or similar references.

The authors also tested the effect of the relieved protein burden for transition to other substrates (page 6, L 185):
"Using the motility deletion strain, we consistently find reduced lag-times for all transitions (Fig. 5B-F)".
However, the bar charts in Figure 5 E do not fully correspond to the data presented in individual growth curves, Figure 5 B,C,D. Maltose growth curves (Figure 5 D) show longest lag for dflhD and somewhat intermediate lag for the WT. The curves for xylose and glycerol show the expected trend with WT having longest lag, but the summary figure 5E does not represent this with WT having intermediate lag time. I suspect that the color code of the points/bars was mixed up. Error bars are missing for lag phase on maltose, while they are present for the other carbon sources.

Throughout the manuscript the authors argue that the expression of acetate metabolism enzymes is non-optimal and leads to unnecessary long lag time. They present two lines of evidence supporting this hypothesis: the additional expression of lacZ dummy protein (increasing lag time), and the deletion of motility functions (reducing lag time). However, both interventions have relatively strong effects on bacterial metabolism including a massive change in protein allocation. It is therefore difficult to pinpoint the addition or removal of idle proteins as the direct cause of the change in lag phase duration; the chain of events is not clear because these interventions can also have other effects, e.g. accelerating translation by freeing up space for ribosomes. It feels as if one key experiment is missing to really prove that the transcript levels for acetate metabolism are non-optimal: the additional expression of aceA/B, malic enzyme, and/or pck. If the expression of these enzymes is really the bottleneck, a constitutive "enzyme reserve" should allow the cells to make a measurably faster transition to growth on acetate. The protein burden of this additional expression would probably lead to lower steady-state growth rate on glucose, illustrating the trade-off between metabolic flexibility and specialization on fast growth. Such an experimental design is explicitly mentioned in the discussion section, page 7 lines 192-197, but was not included here. Is there a reason?

The authors need to be more careful in presentation of their data. In several instances (as pointed out in minor comments) it is not entirely clear what data is shown, how it was transformed, if scales/axes were truncated, if replicate measurements were done, and so on. This can certainly be amended by adding more detail. For example, there is no section about statistics and it is not clear if RNA-Seq measurements were even replicated; Error bars are sometimes drawn and sometimes not. The metric for centrality and dispersion is in most cases not indicated, statistical tests for significance are entirely missing.

Data and model availability: For the sake of transparency, raw data such as the RNA-Seq data should be uploaded to a database if not strong reasons speak against it. The metabolic model is described in a text file and parameters are supplied as table. This is not very practical if other researchers would like to reproduce the modeling results or build upon it. The authors should consider to share the code for their model(s) on a public platform such as github.

Minor points

Figure S1B: Typos, correct "maltate" to malate and "ille" (isoleucine) to ile
Same figure, aceA is indicated as enzyme for malate synthase and isocitrate lyase, while it should be aceA and aceB.
Same figure, labels for malic enzyme maeA/maeB and PEP-carboxykinase, pck, are swapped. Malic enzyme starts from malate, not oxaloacetate.

Figure 1C: the x axis of the RNA-seq results does not correspond to the x-axis and labels given in Figure 1A; in Figure 1A, the lag phase extends about 3.5 hours (also mentioned in text), in 1C it roughly extends from 0 to 130 min, instead of ~200 min. Truncation of the x-axis makes it hard to grasp the true interval between sampling time points. The figure caption does not mention that the axis is not drawn to scale.

Figure 2B,C,D: the x-axis shows relative lacZ expression, with the un-induced condition as reference. The x-axis is scaled from 0 to 4, but it is not clear if this is a "fold change" or a log-scaled fold change. If the data is untransformed: a log2-transformation in Figure 2C would actually show that increase in lacZ and decrease in aceB transcript happens at the same scale (log2 FC of 2 and -2, respectively). Is that coincidence or expected?

Figure S3B: correct "glocolysis"

Figure 4 A,B and Figure 5 all subfigures: The authors do not provide error bars for the RNA-Seq data, nor do they indicate in the figure caption how many replicates were used, or what metric is represented by the points (mean, median?). This makes it difficult to judge the spread and reliability of the data.

Figure 4 C,E,F and text page 5 line 153: Is the increase in steady state growth rate that motility mutants show statistically significant? It certainly looks so for growth on acetate. The authors should consider adding a significance test and p-values. The same applies to measurements of lag time and aceB transcripts.

Dear Dr. Polychronidou,

Thank you for serving as the Senior Editor for our manuscript. We herewith submit a revised version addressing and incorporating the suggestions made by you and the reviewers.

In incorporating the editorial suggestions, we have now separated the introduction and results sections, provided 5 keywords, and included Author Contributions. We also present the "supplementary information" in the *extended view* format, following MSB's guidelines. We include a standfirst text and three bullet points summarizing the key findings in a .doc file named "*standfirst text*" and a "*synopsis*" image file. We have included the methods and materials section in the main text. RNA-seq dataset and the computational model have been made publicly available, with links provided under the Data Availability section of the manuscript. Statistical analysis, number of replicates and the description of the centrality and dispersion are specified in the respective figure captions. The references are included in the style prescribed by MSB, and the author checklist is attached with this submission.

Sincerely,
Jonas Cremer and Rohan Balakrishnan, on behalf of all the authors.

Specific response to reviewer comments:

We appreciate both reviewers' interest in our work and believe that incorporating their suggestions has greatly improved the manuscript. We have particularly performed a key experiment as suggested by the reviewers and included it in the manuscript (Fig. EV1CD). We further added statistical tests to the data presented originally and have made the raw sequencing data and computational models publicly available. Below, we address each of the reviewers' comments in detail. The report of both reviewers is shown in blue.

Reviewer #1:

Balakrishnan et al. presents an interesting study investigating the cause of a lag time during diauxic shift of E. coli. During such a growth transition, cells start to produce enzymes to utilize acetate, and the question is whether cells produce enough of these enzymes to facilitate rapid transition and thereby reduce the lag time. The authors showed that decreasing the expression of a key operon aceBA increases the lag time, and that the same can be achieved by overexpression of an unnecessary gene. These results suggest that there is an inherent competition between the expression of different genes, which the authors modeled theoretically showing that suboptimal allocation of the transcriptome can lead to a prolonged lag time. As some of the most induced genes during the growth transition are flagellum genes that seem unnecessary for the specific environment, the authors created strains lacking such responses and show that the lag time can be reduced from the wildtype.

This is an interesting work showing that the transcriptome response during growth transition may not be optimized for fitness. The data are well presented and support their main model. I have several comments that the authors may consider addressing.

We thank the reviewer very much for the review of our work and the encouraging remarks. We reply to all comments in the following.

1. Does overexpression of aceBA ever reduce the lag time to be shorter than that of the wildtype? The authors used a strong induction system to show that the lag time is sensitive to the level of aceBA. But even at the highest induction level shown, the lag time appears to be still longer than the wildtype one. Is the maximum induction level higher than the level of aceBA in the wildtype? If so, it is interesting, and potentially puzzling, that the increased transcriptional activity of this gene cannot outcompete the resources spent on the other genes to reduce the lag time.

RESPONSE:
We note that lag-times drop strongly below WT values when aceBA is already mildly pre-expressed during steady growth on glucose before glucose runs out. This indicates that aceBA is indeed a major bottleneck limiting growth to resume. We now show these novel experimental results as an important control in Fig EV1CD.
The main point with the result in Fig 1D was to demonstrate that lag-times can decrease simply by increased induction of aceBA genes at the moment of glucose depletion. We modify lines 82-86 in the main text to better describe this point. But it is indeed notable that lag-times do not fall substantially below WT levels when titrating the expression of aceBA at the start of the shift once glucose runs out. We think this has to do with the relatively weak expression of the chromosomal construct we used to titrate aceBA; the native aceBA promoter strongly upregulates aceBA expression (20-30 fold increase in acetate, PMID: 11815613). It is thus likely that our chromosome based titration construct may not go beyond WT expression levels while a stronger expression could further shorten lag-times. In an attempt to further increase aceBA expression we generated a new strain which harbors these genes on a high-copy number plasmid. Unfortunately, such a strain did not allow for a clean experiment as we observe greatly reduced lag times even without any induction, most likely due to leaky expression stemming from the high-copy plasmid system (Response figure 1). The new strain also showed slower growth during pre-shift growth on glucose, further complicating interpretations. We thus refrained from using the results from the plasmid construct and only show the pre-expression results to establish that aceBA is indeed a major bottleneck which limits growth to resume (new Fig. EV1CD).

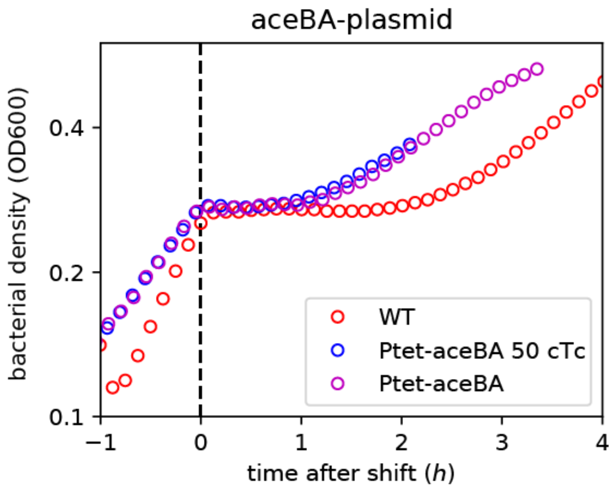

Response figure 1: Growth transitions from growth on glucose to growth on acetate. Lag times observed for WT cells and cells harboring Ptet-aceBA on a high-copy number plasmid to titrate aceB expression at the moment of glucose depletion.

2. The theoretical model makes quantitative predictions on how much lag time should be observed at a given allocation to the required enzymes (Fig 3B). I was surprised that there was no comparison between experimental data and this prediction, even though the data

already exist in Figure 4. The model feels unnecessary if the quantitative predictions are not tested.
RESPONSE: We followed the suggestion and now directly compare the model predictions with observed changes in lag-times. To allow a comparison with minimal fitting we specifically analyzed how the model describes lag-time changes when lacZ as useless protein is expressed at different levels during the shift. The model captures the effect of reduced AceB expression on lag times resulting from increased lacZ titration without free fitting. We show these results in the novel figure panel Fig. 3C. We discuss the comparison and fitting procedures in detail in the caption of Fig. 3 and the Appendix Text 1.

3. The 'wildtype' E. coli exists in many different motility genotypes, with many different mutations that stimulate flagellum genes to different degrees (https://journals.asm.org/doi/10.1128/JB.00455-15). It would be interesting to explore whether the strain used in this study has the highest level of unnecessary motility gene expression during diauxic shift. If most wildtype isolates behave similarly, the results could strengthen the claim that the response is not optimized for the growth transition.

RESPONSE: We agree that exploring the diauxic behavior versus gene-expression patterns in wild isolates is highly interesting and this is indeed the subject of ongoing studies in JC's group. Different diauxic behavior may originate not just from differences in the expression of motility genes but also from the differential expression of carbon transporters genes. While not a part of the current manuscript, we would like to share Response figure 2 with the reviewer, showing that E. coli cells isolated recently from the human gut (PMID: 6363394) exhibit a wide range of glucose to acetate lag-times, that are both, greatly longer and shorter than the reference strain (NCM3722, Resp. fig. 2 blue bar) used in this study. We plan to explore this variation systematically in the context of the hard-wired "diversifying responses" discussed in this manuscript. But since a careful strain comparison requires an in-depth physiology characterization of each strain we think that such a study goes well beyond the scope of the manuscript we presented here.

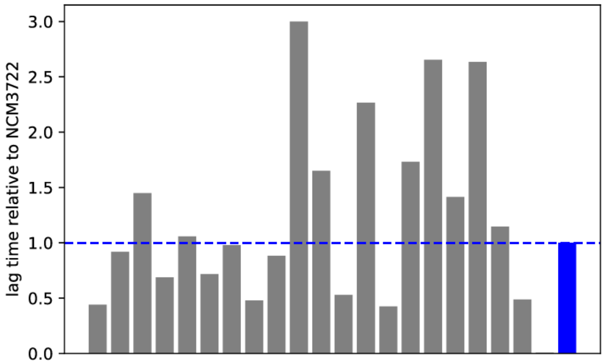

Response figure 2: Variability of glucose-acetate lag-times among different E. coli strains isolated from the human gut microbiota. The difference in lag-times for isolates are shown in comparison to NCM3722 (blue bar), the WT strain used in this work.

4. In the abstract, the sentence that "our findings highlight that cells do not optimize growth in the given environment ..." seems somewhat misleading. The focus of this study is at changing environments and not a given environment. The former is history-dependent whereas the latter implies a steady-state that is history-free. I suggest changing "in the given environment" to "under changing environments."
RESPONSE: The suggested edit is more accurate and we have incorporated it in the abstract.

Reviewer #3:

Summary

The study by Balakrishnan and co-authors sheds light on the question why E. coli requires a several hour long lag-phase to make the transition from a high nutrient quality substrate, glucose, to a less-preferred substrate, acetate. Using knock-out and overexpression strains, transcriptional profiling, and resource allocation modeling, the authors show that this transition is not growth-optimal due to a diversified gene expression program. This program prevents the timely production of required enzymes and leads to a long lag phase. The lag phase can be tweaked by adding or removing the expression of genes that result in "idle" proteins.

General remarks

This study advances our knowledge of growth shifts by highlighting the importance of timely production of the required enzymes, and how other cell functions drain resources from production of these enzymes. The study adds more evidence for the hypothesis that bacteria are evolutionarily optimized for metabolic flexibility, e.g. by prioritizing nutrient scavenging (motility) over quick transition towards a specific carbon source. The study is highly interesting, well-written and experiments are sound and support the drawn conclusions. However, there are also several weaknesses that should be addressed: The authors might consider to add one particular experiment in order to prove that acetate metabolism transcripts are really the limiting factor for lag phase duration (see major comments). There are problems with some figures where the conclusions do not match the presented data (most likely because of trivial errors). Generally, the level of detail for reporting data and statistical methods is below standard and can certainly be improved by addressing some of the comments outlined below.

We thank the reviewer very much for the review of our work and the encouraging remarks. We have adjusted our manuscript to address all points and concerns raised by the reviewer and reply in detail in the following.

Major points

P3, l68: The authors write that "Lag times are greatly reduced when increasing amounts of the inducer chlorotetracycline (cTc) are added at the moment of glucose depletion (Figure 1D)." This statement is somewhat misleading, as the lag time at full induction (100 ng/mL cTc) seems to be identical with the lag time when using the natural ace promoter, about 3.5 h. Judging from figure 1D, it is not obvious that lag time can be reduced, but it can certainly be prolonged. The authors need to clarify what the reference is when stating that lag time can be reduced, particularly because this is used to motivate the subsequent study of resource allocation during the lag phase.

RESPONSE: We thank the reviewer for pointing out the lack of clarity in the description of figure 1D and have modified the text to better describe that the reduction in lag time upon the highest induction of aceBA is in comparison to the case with no induction.

This line now reads as follows: “*In this strain, as increasing concentrations of the inducer chlorotetracycline (cTc) is added at the moment of glucose depletion, growth recovery is progressively faster, from no recovery for over 10 hours in the absence of induction to ~3 hr recovery at the highest cTc concentration used. (**Figure 1D**).*”

In Figure 2 and in text page 3, line 82+, the authors nicely show that lag phase increases proportionally with lacZ expression, and aceA/B down-regulation. The authors attribute the lag-phase mechanistically to the reduced transcript concentration of acetate-metabolizing enzymes. However, is it not possible that the increased lag phase is a product of several overlaying effects all originating from lacZ-expression? With the assumption that protein pool size is constant but limited, we can think of:
- more lacZ transcripts, less ace transcripts (explicitly mentioned)
RESPONSE: In principle, many potential effects of lacZ expression could influence lag-times. In our manuscript we explicitly mention the "*more lacZ, less ace transcripts*" scenario because (a) we demonstrated the importance of aceBA expression on lag times (figure 1D); (b) we showed that aceBA mRNAs decrease as lacZ mRNA is dialed up (figure 2CD); and (c) the simple assumption that mRNA abundance sets up the competition for all other resources like ribosomes and amino acid precursors, especially if protein pool size is considered constant, as the reviewer posits.
- lacZ dummy protein reducing ribosomal proteins needed to translate acetate metabolism enzymes (not explicitly mentioned)
- lacZ dummy protein reducing other metabolic enzymes apart from acetate metabolism (not explicitly mentioned)
Maybe the authors can clarify if and how these indirect effects of lacZ expression could explain the observed results, or how they can be untangled.

RESPONSE: During the growth arrest, there is no significant increase in the protein content owing to the reduced overall protein synthesis (PMID: 29072300). Hence, the abundance of the newly made dummy protein would not significantly reduce the abundance of the ribosomes and other metabolic proteins that were already synthesized by the cell before the growth arrest. The strongest effect of the dummy-gene expression would thus be the reduced allocation of cellular resources towards the newly required genes, and not reduced abundances of these resources themselves. The alternate scenarios raised by the reviewer will however become very crucial later during the shift when growth resumes on acetate. Growth rate on acetate will indeed be slower due to the dummy protein accumulation, as can for example be seen in the post-shift slopes in figure 2A. But, to keep the focus on how the resource allocation during the cellular response influences recovery times, we avoided discussing them.

The authors show that deletion of "idle" proteins (motility functions) leads to increased growth rate and decreased lag phase during acetate transition. Page 5, line 158 reads: "The increase of growth was also observed for other carbon sources besides acetate (Fig. S6) and provides direct support for the idea that gene regulation is not optimized for steady state growth."
I just want to point out that non-optimality of gene expression for steady-state growth was also shown in other organisms by now, apart from E. coli. For example, streamlining a Pseudomonas putida strain by deleting motility genes increased growth rate and stress tolerance (Martinez-Garcia et al., Env Microb, 2013). In photosynthetic cyanobacteria, the light harvesting machinery was identified as a major protein burden that is idle in high light conditions, leading to non-optimal steady state growth in this condition (Jahn et al., Cell Reports, 2018. Full disclosure: I am an author of this paper). The authors may consider including these or similar references.

RESPONSE: We thank the reviewer for alerting us to these pertinent studies in other bacterial systems. We think that the inclusion of these works (lines 181-185) strengthens the message on the non-optimality of bacterial gene expression.

The authors also tested the effect of the relieved protein burden for transition to other substrates (page 6, L 185):
"Using the motility deletion strain, we consistently find reduced lag-times for all transitions (Fig. 5B-F)".
However, the bar charts in Figure 5 E do not fully correspond to the data presented in individual growth curves, Figure 5 B,C,D. Maltose growth curves (Figure 5 D) show longest lag for dflhD and somewhat intermediate lag for the WT. The curves for xylose and glycerol show the expected trend with WT having longest lag, but the summary figure 5E does not represent this with WT having intermediate lag time. I suspect that the color code of the points/bars was mixed up. Error bars are missing for lag phase on maltose, while they are present for the other carbon sources.
RESPONSE: We thank the reviewer for pointing out these discrepancies. Embarrassingly enough, these problems were indeed traced to inconsistency in color coding. We have now fixed this, along with the issue of the missing error bars for maltose in an updated figure 5.

Throughout the manuscript the authors argue that the expression of acetate metabolism enzymes is non-optimal and leads to unnecessary long lag time. They present two lines of evidence supporting this hypothesis: the additional expression of lacZ dummy protein (increasing lag time), and the deletion of motility functions (reducing lag time). However, both interventions have relatively strong effects on bacterial metabolism including a massive change in protein allocation. It is therefore difficult to pinpoint the addition or removal of idle proteins as the direct cause of the change in lag phase duration; the chain of events is not clear because these interventions can also have other effects, e.g. accelerating translation by freeing up space for ribosomes. It feels as if one key experiment is missing to really prove that the transcript levels for acetate metabolism are non-optimal: the additional expression of aceA/B, malic enzyme, and/or pck. If the expression of these enzymes is really the bottleneck, a constitutive "enzyme reserve" should allow the cells to make a measurably faster transition to growth on acetate. The protein burden of this additional expression would probably lead to lower steady-state growth rate on glucose, illustrating the trade-off between metabolic flexibility and specialization on fast growth. Such an experimental design is explicitly mentioned in the discussion section, page 7 lines 192-197, but was not included here. Is there a reason?

RESPONSE: We agree with the reviewer about the need for demonstrating that *aceBA* expression is really the bottleneck. As suggested by the reviewer, we added a new experiment and pre-expressed the aceBA genes before glucose runs out. We observed indeed that maintaining these "*enzyme reserves*" reduces the lag-times (Response Fig. 3). Lag-times strongly decrease compared to WT cells. These new findings are now shown in Fig. EV1CD. Notably, this reduction in lag times is seen even when the aceBA pre-expression levels are mild-enough to not slow the growth on glucose (Response figure 3C, pre shift growth rates are comparable between non-induced and induced cultures).

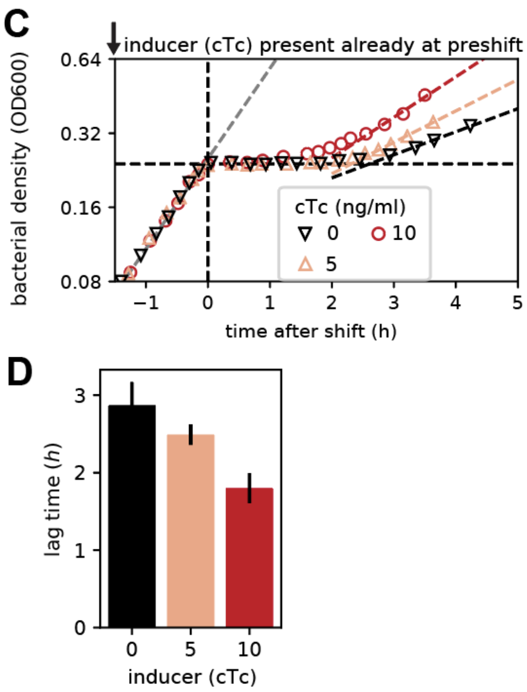

Response figure 3: Effect of maintaining pre-expressed aceBA reserves on growth transitions. Diauxic transitions (top panel) of NQ1350 in which the native aceBA promoter is replaced by the inducible ptet promoter is shown when 0, 5 or 10 ng/ml inducer cTc.

The authors need to be more careful in presentation of their data. In several instances (as pointed out in minor comments) it is not entirely clear what data is shown, how it was transformed, if scales/axes were truncated, if replicate measurements were done, and so on. This can certainly be amended by adding more detail. For example, there is no section about statistics and it is not clear if RNA-Seq measurements were even replicated; Error bars are sometimes drawn and sometimes not. The metric for centrality and dispersion is in most cases not indicated, statistical tests for significance are entirely missing.
RESPONSE: We appreciate the critique and have adjusted and improved the overall data presentation throughout the main and extended view figures. We now clearly state the quantities plotted (mean and standard deviations) and the number of independent biological replicates tested. We include tests for statistical significance in each figure caption where applicable. See also our reply below to the detailed points raised by the reviewer.

Data and model availability: For the sake of transparency, raw data such as the RNA-Seq data should be uploaded to a database if not strong reasons speak against it. The metabolic model is described in a text file and parameters are supplied as table. This is not very practical if other researchers would like to reproduce the modeling results or build upon it. The authors should consider to share the code for their model(s) on a public platform such as github.
RESPONSE: We are committed to provide easy access to data and modeling code. RNA-seq data is now available at the GEO database (accession number GSE185426) and the metabolic model is available via GitHub (link) as suggested by the reviewer.

## Minor points
Figure S1B: Typos, correct "maltate" to malate and "ille" (isoleucine) to ile
Same figure, aceA is indicated as enzyme for malate synthase and isocitrate lyase, while it should be aceA and aceB.
Same figure, labels for malic enzyme maeA/maeB and PEP-carboxykinase, pck, are swapped.

Malic enzyme starts
RESPONSE: We thank the reviewer identifying these errors and have corrected each of these.

Figure 1C: the x axis of the RNA-seq results does not correspond to the x-axis and labels given in Figure 1A; in Figure 1A, the lag phase extends about 3.5 hours (also mentioned in text), in 1C it roughly extends from 0 to 130 min, instead of ~200 min. Truncation of the x-axis makes it hard to grasp the true interval between sampling time points. The figure caption does not mention that the axis is not drawn to scale.
RESPONSE: Following the reviewer's remark the x-axis now shows hours to make it comparable to figure 1A. We improved the figure to better indicate the break in the x-axis, which we now also explicitly describe in the figure caption.

Figure 2B,C,D: the x-axis shows relative lacZ expression, with the un-induced condition as reference. The x-axis is scaled from 0 to 4, but it is not clear if this is a "fold change" or a log-scaled fold change. If the data is untransformed: a log2-transformation in Figure 2C would actually show that increase in lacZ and decrease in aceB transcript happens at the same scale (log2 FC of 2 and -2, respectively). Is that coincidence or expected?
RESPONSE: We update this panel by explicitly labelling the axes as "fold-changes" to avoid confusion. The raised point about the scaling of expression of lacZ and aceB is interesting. In the updated Fig. 2C, we now also show the fold-changes on a log2-scale as per the reviewer's comment. The "matching of scales" appears to be in agreement with the simple picture that the expression of all genes (including aceB) are uniformly affected when overexpressing lacZ. But we avoid stressing this statement in the manuscript given that it would be based on qPCR data for only two mRNAs, aceB (Fig 2B-D) and aceA (Fig. EV2).

Figure S3B: correct "glocolysis"
RESPONSE: We corrected the typo.

Figure 4 A,B and Figure 5 all subfigures: The authors do not provide error bars for the RNA-Seq data, nor do they indicate in the figure caption how many replicates were used, or what metric is represented by the points (mean, median?). This makes it difficult to judge the spread and reliability of the data.
RESPONSE: In this work, we performed RNA-seq to guide our other experiments by identifying potential "non-required" genes that we could delete. We thus performed it only once per time-point during the shift. We now clarify this in the captions of Figure 1 and 4. Notably however, the multiple data points collected through the time-course of the transition (5, 15, 60, 120 minutes) show very consistent trends of gene expression suggesting a high level of reliability, at least when considering the relative abundance of highly expressed genes or groups of genes.

Figure 4 C,E,F and text page 5 line 153: Is the increase in steady state growth rate that motility mutants show statistically significant? It certainly looks so for growth on acetate. The authors should consider adding a significance test and p-values. The same applies to measurements of lag time and aceB transcripts.
RESPONSE: To test for significance we performed T-tests and now provide p-values where appropriate (captions of figures 4, 5, and EV6). The analysis confirms that differences in lag-times and expression (figures 4EF and 5) are significant. Differences in growth-rates are also

significant for growth on acetate (Figure 4C). To further promote transparency, individual measurements and the derived means, standard deviations, and p-values are now available in the source data file we provide.

Manuscript Number: MSB-2021-10597R
Title: Suboptimal resource allocation in changing environments constrains response and growth in bacteria

Thank you for sending us your revised manuscript. We have now heard back from the two reviewers who were asked to evaluate your revised study. As you will see below, the reviewers are satisfied with the performed revisions and are supportive of publication. As such, I am glad to inform you that we can soon proceed with formally accepting the study for publication, pending some minor editorial issues, which we would ask you to address in a minor revision.

Reviewer #1:

The authors have addressed my comments. I recommend publication of this interesting work.

Reviewer #3:

The authors have done excellent work to improve the manuscript. All comments and criticism have been addressed sufficiently.

The authors performed the requested editorial changes.

Thank you again for sending us your revised manuscript. We are now satisfied with the modifications made and I am pleased to inform you that your paper has been accepted for publication.

EMBO PRESS

YOU MUST COMPLETE ALL CELLS WITH A PINK BACKGROUND ↓

PLEASE NOTE THAT THIS CHECKLIST WILL BE PUBLISHED ALONGSIDE YOUR PAPER

Corresponding Author Name: Jonas Cremer, Rohan Balakrishnan

Journal Submitted to: Molecular Systems Biology
Manuscript Number: MSB-2021-10597

Reporting Checklist For Life Sciences Articles (Rev. June 2017)

This checklist is used to ensure good reporting standards and to improve the reproducibility of published results. These guidelines are consistent with the Principles and Guidelines for Reporting Preclinical Research issued by the NIH in 2014. Please follow the journal's authorship guidelines in preparing your manuscript.

A- Figures

1. Data

The data shown in figures should satisfy the following conditions:

- the data were obtained and processed according to the field's best practice and are presented to reflect the results of the experiments in an accurate and unbiased manner.
- figure panels include only data points, measurements or observations that can be compared to each other in a scientifically meaningful way.
- graphs include clearly labeled error bars for independent experiments and sample sizes. Unless justified, error bars should not be shown for technical replicates.
- if n< 5, the individual data points from each experiment should be plotted and any statistical test employed should be justified
- Source Data should be included to report the data underlying graphs. Please follow the guidelines set out in the author ship guidelines on Data Presentation.

2. Captions

Each figure caption should contain the following information, for each panel where they are relevant:

- a specification of the experimental system investigated (eg cell line, species name).
- the assay(s) and method(s) used to carry out the reported observations and measurements
- an explicit mention of the biological and chemical entity(ies) that are being measured.
- an explicit mention of the biological and chemical entity(ies) that are altered/varied/perturbed in a controlled manner.

- the exact sample size (n) for each experimental group/condition, given as a number, not a range;
- a description of the sample collection allowing the reader to understand whether the samples represent technical or biological replicates (including how many animals, litters, cultures, etc.).
- a statement of how many times the experiment shown was independently replicated in the laboratory.
- definitions of statistical methods and measures:
  - common tests, such as t-test (please specify whether paired vs. unpaired), simple χ^2 tests, Wilcoxon and Mann-Whitney tests, can be unambiguously identified by name only, but more complex techniques should be described in the methods section;
  - are tests one-sided or two-sided?
  - are there adjustments for multiple comparisons?
  - exact statistical test results, e.g., P values = x but not P values < x;
  - definition of 'center values' as median or average;
  - definition of error bars as s.d. or s.e.m.

Any descriptions too long for the figure legend should be included in the methods section and/or with the source data.

In the pink boxes below, please ensure that the answers to the following questions are reported in the manuscript itself. Every question should be answered. If the question is not relevant to your research, please write NA (non applicable). We encourage you to include a specific subsection in the methods section for statistics, reagents, animal models and human subjects.

USEFUL LINKS FOR COMPLETING THIS FORM

http://www.antibodypedia.com
http://1degreebio.org
http://www.equator-network.org/reporting-guidelines/improving-bioscience-research-repor
http://grants.nih.gov/grants/olaw/olaw.htm
http://www.mrc.ac.uk/Ourresearch/Ethicsresearchguidance/Useofanimals/index.htm
http://ClinicalTrials.gov
http://www.consort-statement.org
http://www.consort-statement.org/checklists/view/32-consort/66-title
http://www.equator-network.org/reporting-guidelines/reporting-recommendations-for-tumo
http://datadryad.org
http://figshare.com
http://www.ncbi.nlm.nih.gov/gap
http://www.ebi.ac.uk/ega
http://biomodels.net/
http://biomodels.net/miriam/
http://jjj.biochem.sun.ac.za
https://osp.od.nih.gov/biosafety-biosecurity-and-emerging-biotechnology/
http://www.selectagents.gov/

B- Statistics and general methods

Please fill out these boxes ↓ (Do not worry if you cannot see all your text once you press return)

1.a. How was the sample size chosen to ensure adequate power to detect a pre-specified effect size?	All experiments were performed with E. coli growing in batch cultures with cell densities (OD600 of 0.1 to 0.4) that are well within the linear detection range of our spectrophotometers. Culture volumes were chosen to allow for up to 50 OD measurements and approximately 500ng RNA extraction (a concentration at which qPCR or RNA-seq is independent of sample size).
1.b. For animal studies, include a statement about sample size estimate even if no statistical methods were used.	NA
2. Describe inclusion/exclusion criteria if samples or animals were excluded from the analysis. Were the criteria pre-established?	No samples were excluded from the analysis.
3. Were any steps taken to minimize the effects of subjective bias when allocating animals/samples to treatment (e.g. randomization procedure)? If yes, please describe.	NA
For animal studies, include a statement about randomization even if no randomization was used.	NA
4.a. Were any steps taken to minimize the effects of subjective bias during group allocation or/and when assessing results (e.g. blinding of the investigator)? If yes please describe.	No
4.b. For animal studies, include a statement about blinding even if no blinding was done	NA
5. For every figure, are statistical tests justified as appropriate?	Captions for every figure describes the centrality (means), dispersion (Standard deviations), independent biological replicate numbers, and p values, whenerver applicable. Biological repeats follow standard distributions well, justifying the used tests.
Do the data meet the assumptions of the tests (e.g., normal distribution)? Describe any methods used to assess it.	We do not expect our estimates of growth rates and lag-times that use spectroscopic measurements at OD600 to carry any bias. Similarly, we also do not observe any bias in qPCR measurements for the primer-sets employed in this study. We therefore expect all data to follow a standard distrubtion around ther mean value.

Is there an estimate of variation within each group of data?	Where applicable we provide standard deviations and the tumber of independent biological replicates performed.
Is the variance similar between the groups that are being statistically compared?	Yes

C- Reagents

6. To show that antibodies were profiled for use in the system under study (assay and species), provide a citation, catalog number and/or clone number, supplementary information or reference to an antibody validation profile. e.g., Antibodypedia (see link list at top right), 1DegreeBio (see link list at top right).	No antibodies were used in this work.
7. Identify the source of cell lines and report if they were recently authenticated (e.g., by STR profiling) and tested for mycoplasma contamination.	Information on the Escherichia coli strain used in this work is given in the methods section. Strain identity have been confirmed by sequencing.

* for all hyperlinks, please see the table at the top right of the document

D- Animal Models

8. Report species, strain, gender, age of animals and genetic modification status where applicable. Please detail housing and husbandry conditions and the source of animals.	No animal models were used in this work
9. For experiments involving live vertebrates, include a statement of compliance with ethical regulations and identify the committee(s) approving the experiments.	No animal models were used in this work
10. We recommend consulting the ARRIVE guidelines (see link list at top right) (PLoS Biol. 8(6), e1000412, 2010) to ensure that other relevant aspects of animal studies are adequately reported. See author guidelines, under 'Reporting Guidelines'. See also: NIH (see link list at top right) and MRC (see link list at top right) recommendations. Please confirm compliance.	No animal models were used in this work

E- Human Subjects

11. Identify the committee(s) approving the study protocol.	No human subjects were used in this work
12. Include a statement confirming that informed consent was obtained from all subjects and that the experiments conformed to the principles set out in the WMA Declaration of Helsinki and the Department of Health and Human Services Belmont Report.	No human subjects were used in this work
13. For publication of patient photos, include a statement confirming that consent to publish was obtained.	No human subjects were used in this work
14. Report any restrictions on the availability (and/or on the use) of human data or samples.	No human subjects were used in this work
15. Report the clinical trial registration number (at ClinicalTrials.gov or equivalent), where applicable.	No human subjects were used in this work
16. For phase II and III randomized controlled trials, please refer to the CONSORT flow diagram (see link list at top right) and submit the CONSORT checklist (see link list at top right) with your submission. See author guidelines, under 'Reporting Guidelines'. Please confirm you have submitted this list.	No human subjects were used in this work
17. For tumor marker prognostic studies, we recommend that you follow the REMARK reporting guidelines (see link list at top right). See author guidelines, under 'Reporting Guidelines'. Please confirm you have followed these guidelines.	No human subjects were used in this work

F- Data Accessibility

18: Provide a "Data Availability" section at the end of the Materials & Methods, listing the accession codes for data generated in this study and deposited in a public database (e.g. RNA-Seq data: Gene Expression Omnibus GSE39462, Proteomics data: PRIDE PXD000208 etc.) Please refer to our author guidelines for 'Data Deposition'.<br><br>Data deposition in a public repository is mandatory for:<br>a. Protein, DNA and RNA sequences<br>b. Macromolecular structures<br>c. Crystallographic data for small molecules<br>d. Functional genomics data<br>e. Proteomics and molecular interactions	Data availability section has been included in the revised submission.
19. Deposition is strongly recommended for any datasets that are central and integral to the study; please consider the journal's data policy. If no structured public repository exists for a given data type, we encourage the provision of datasets in the manuscript as a Supplementary Document (see author guidelines under 'Expanded View' or in unstructured repositories such as Dryad (see link list at top right) or Figshare (see link list at top right).	We have deposited the RNA seq raw data in Gene Expression Omnibus (GEO), accession number GSE185426.
20. Access to human clinical and genomic datasets should be provided with as few restrictions as possible while respecting ethical obligations to the patients and relevant medical and legal issues. If practically possible and compatible with the individual consent agreement used in the study, such data should be deposited in one of the major public access-controlled repositories such as dbGAP (see link list at top right) or EGA (see link list at top right).	NA
21. Computational models that are central and integral to a study should be shared without restrictions and provided in a machine-readable form. The relevant accession numbers or links should be provided. When possible, standardized format (SBML, CellML) should be used instead of scripts (e.g. MATLAB). Authors are strongly encouraged to follow the MIRIAM guidelines (see link list at top right) and deposit their model in a public database such as Biomodels (see link list at top right) or JWS Online (see link list at top right). If computer source code is provided with the paper, it should be deposited in a public repository or included in supplementary information.	The computational code of the model is available via Github at https://github.com/jonascremer/lagtimemodeling. Data shown in Figures is available in a joint source data file.

G- Dual use research of concern

22. Could your study fall under dual use research restrictions? Please check biosecurity documents (see link list at top right) and list of select agents and toxins (APHIS/CDC) (see link list at top right). According to our biosecurity guidelines, provide a statement only if it could.	The study does not fall under dual research restrictions
